# RORγt agonist enhances anti-PD-1 therapy by promoting monocyte-derived dendritic cells through CXCL10 in cancers

**DOI:** 10.1186/s13046-022-02289-2

**Published:** 2022-04-23

**Authors:** Li Xia, Enming Tian, Mingcheng Yu, Chenglong Liu, Lian Shen, Yafei Huang, Zhongen Wu, Jinlong Tian, Ker Yu, Yonghui Wang, Qiong Xie, Di Zhu

**Affiliations:** 1 Department of Pharmacology, School of Pharmacy, Fudan University, Shanghai, 201203 China; 2 Department of Pharmacology, School of Basic Medical Sciences, Fudan University, Shanghai, 200032 China; 3 Department of Medicinal Chemistry, School of Pharmacy, Fudan University, Shanghai, 201203 China

**Keywords:** RORγt, Th17, CXCL10, Tumor microenvironment, Immunotherapy

## Abstract

**Background:**

The overall response rate to checkpoint blockade remains unsatisfactory, partially due to the immune-suppressive tumor microenvironment. A retinoic acid-related orphan receptor γt (RORγt) agonist (LYC-55716) is currently used in clinical trials combined with anti-PD-1, but how the Th17 cell transcription factor RORγt enhances antitumor immunity of PD-1 in the tumor microenvironment remains elusive.

**Methods:**

The expression of mRNA was analyzed using qPCR assays. Flow cytometry was used to sort and profile cells. Cell migration was analyzed using Transwell assays. Biacore was used to determine the binding affinity to the RORγt protein. The RORγt GAL4 cell-based reporter gene assay was used to measure activity in the RORγt driven luciferase reporter gene expression.

**Results:**

We designed a potent and selective small-molecule RORγt agonist (8-074) that shows robust antitumor efficacy in syngeneic tumor models and improves the efficacy of anti‑PD‑1 in a murine lung cancer model. RORγt agonist treatment increased intratumoral CD8^+^ T cells, which were correlated with CXCL10 and monocyte-derived dendritic cells (MoDCs). In addition, the RORγt agonist promoted Type 17 T cell migration by upregulating *CCL20* and *CCR6* expression, and Type 17 T cell tumor infiltration. CCL20 induces MoDCs migration, and CXCL10 derived from MoDCs promotes CD8^+^ T cell migration.

**Conclusion:**

Our results revealed that the RORγt agonist improved the efficacy of anti-PD-1. The RORγt agonist increased the migration of MoDCs, which increased the local levels of CXCL10, thus promoting CD8^+^ T cell tumor infiltration. Our findings provide the mechanistic insights implicating the RORγt agonist in immunotherapy and offer a strategy for targeting the RORγt agonist to improve PD-1 antibody efficacy in cancers.

**Supplementary Information:**

The online version contains supplementary material available at 10.1186/s13046-022-02289-2.

## Background

Retinoic acid-related orphan receptor γ (RORγ) is a target for both anti-cancer and anti-inflammation drugs. RORγt is a thymus-specific isoform of RORγ that plays a crucial role in the differentiation of Type 17 T cells, including CD4^+^ helper T cells (Th17) and CD8^+^ cytotoxic T cells (Tc17) in humans and mice [1]. In addition, as a master transcription factor, RORγt promotes the differentiation of IL-17-expressing innate immune cell subpopulations (namely, Th17 cells, Tc17 cells, NK cells, and γδT cells), regulates the survival of T cells, and activates Th17 and Tc17 cells to secrete effector cytokines such as IL-17A, IL-17F, GM-CSF, and IL-22 [2, 3].

Interleukin 17A (IL-17A), as a hallmark cytokine of Type 17 T cells, has antitumor effects depending on the tumor environment and tumor type [1]. RORγt^+^ Type 17 T cells and their signature cytokine IL-17A have also been associated with enhanced antitumor effects [4]. It has been reported that IL-17A exhibits antitumor effects during tumor occurrence and metastasis, acting as a prognostic biomarker [5, 6]. Type 17 T cells can mediate potent and durable tumor growth inhibition when transferred to tumor-bearing animals [7–9]. On the one hand, Tc17 has more survival advantages and superior direct cytotoxicity compared to Tc1 cells [9]; Type 17 T cells secrete IL-17, GM-CSF, and IFN-γ to recruit immune cells such as T cells, B cells, granulocytes, and macrophages to the tumor tissue [9–11]. Moreover, IL-17 produced by Type 17 T cells can also play an antitumor role by activating cytotoxic T lymphocytes (CTL) and natural killer cells (NK) [6, 12].

Type 17 T cells and their effector cytokines play an important role in tumor immunity. Synthetic RORγt agonists can regulate the gene expression of effector cytokines to enhance Type 17 T cell effector function and modulate the tumor microenvironment (TME) by increasing the immune activity and decreasing immune suppression at the same time [9, 13]. A tertiary amine RORγt agonist (JG-1) was discovered in the dual fluorescent resonance energy transfer (dual FRET) assay (EC_50_: 20 nM) [14]. However, the molecular activity of JG-1 is not sufficient to trigger a cellular response [15]. Based on the co-crystallography structure of JG-1 and the ligand binding domain (LBD) of RORγt, a novel RORγt agonist (8b) with improved cellular activity (EC_50_: 37.2 nM) to promote the IL-17A level in  vitro was identified as a potential lead compound [15]. Feng et al. reported a triterpenoid RORγt agonist with the EC_50_ at 11.4 nM binding to RORγt in a thermo shift assay [16]. Researchers at the Scripps Institute found a series of N-benzyl indolines modulators that exhibited good RORγt agonism activity with EC_50_ at 30 nM [17]. Takeda Pharmaceuticals disclosed a series of N-benzyl indolines modulators that exhibited strong RORγt agonism. Compound D in the fluorescent resonance energy transfer (FRET)-RORγt SRC1 assay has an EC_50_ at 4.1 nM [18]. Ma et al. found a novel N-sulfonamide-tetrahydroisoquinoline as a potent RORγt agonist, and compound 28 showed an EC_50_ of 21 nM in the dual FRET assay and in mouse Th17 cell differentiation [18]. LYC-55716, an oral agonist of RORγt, was discovered by Lycera. A Phase I/II trial of LYC-55716 is ongoing to treat adult patients with relapsed or refractory metastatic solid tumors who failed to respond to standard therapies [19]. Phase I clinical results with LYC-55716 identified a pharmacodynamically active dose and showed that this agent was well tolerated in patients [19]. A Phase IIa expansion trial of LYC-55716 in patients with selected solid tumors (NCT02929862) was completed. In addition, a Phase Ib study of LYC-55716 and pembrolizumab in patients with non-small cell lung cancer is ongoing (NCT03396497).

A high density of tumor-infiltrating lymphocytes was reported to be associated with favorable clinical outcomes in various cancer types. CTL infiltration to the tumor site is essential for effective immunotherapy [20]. However, the mechanism underlying immune cells infiltration in Lewis lung carcinoma (LLC) tumor tissues mediated by RORγt agonist or IL-17A is not fully understood [21]. In our study, we uncovered the role of Type 17 T cells in the regulation of CD8^+^ T cell tumor infiltration using a novel small molecule, the RORγt synthetic agonist named 8-074, in an LLC model. We found that 8-074 facilitated cytokine production by Type 17 T cells to modulate the TME, and the chemokine upregulation attracted immune cells to the tumor site, resulting in potent antitumor responses.

## Methods and materials

### Cell culture and chemicals

The cell lines MC38, LLC, B16F10, and EL4 (from the American type culture collection and identified by the Shanghai Yihe Biological Company) were cultured according to the supplier’s recommendations. The cells were cultured in Dulbecco’s modified Eagle’s medium (DMEM, HyClone, Logan, Utah) supplemented with 10% fetal bovine serum (FBS, Gibco, California, USA) and 1% penicillin/streptomycin (Gibco, California, USA). The second passage of the cells was used. All the cells were kept at 37 °C and cultured in a 5% CO_2_ cell incubator.

### Animal source

Wild-type C57BL/6 mice were purchased from the Beijing Vital River Laboratory Animal Technology Co., Ltd. (Shanghai, China). Females were 16 - 18 g and 6 - 8 weeks old. OT-I mice, CD45.1 mice, and CD45.2 mice were purchased from the Southern Model Biotechnology Co., Ltd. (Shanghai, China). Mice carrying the CD45.1 gene were mated with the OT-I mice to obtain CD45.1 OT-I double-positive mice. All mice were raised in Specific Pathogen Free (SPF) (license ID: SYXK(Shanghai)2020-0032). All the animal experiments were conducted in accordance with the U.K. Animals (Scientific Procedures) Act of 1986 and the associated guidelines, as well as the EU Directive 2010/63/EU for animal experiments. All animal studies complied with the ARRIVE guidelines.

### Mouse type 17 cell differentiation

CD4^+^ CD25^−^CD62L^high^ cells were purified from C57BL/6 splenocytes using an EasySep Mouse Naïve CD4^+^ T cell isolation kit from STEMCELL Technologies (Vancouver, Canada), and they were differentiated into Th17 cells (TGF-β, 2 ng/ml; IL-6, 20 ng/ml; Anti-IFN-γ, 10 μg/ml; Anti-IL-4, 10 μg/ml, BioLegend, San Diego, CA) in the presence of plate-bound anti-CD3 (5 μg/ml, BioLegend, San Diego, CA) and anti-CD28 (2 μg/ml, BioLegend, San Diego, CA). The cells were harvested and processed for cytokine analysis at the RNA or protein level using real-time qPCR, flow cytometry, and ELISA on day five. Alternatively, splenocytes from OT-I mice were activated using OVA-derived peptides SIINFEKL (50 ng/ml, Sangon Biotech, Shanghai, China) and polarized to Tc17 cells using cytokine TGF-β (2 ng/ml, BioLegend, San Diego, CA) and IL-6 (20 ng/ml, BioLegend, San Diego, CA) for four or five days.

### Human type 17 T cell differentiation

Human PBMCs were donated by Li Xia, who provided her written informed consent. All the cells were used in vitro only. The collection of human PBMCs was approved by the ethics committee of the Fudan Affiliated Minhang Hospital (2019-Pijian-010-01 K). Whole human blood was obtained from healthy volunteers, and peripheral blood mononuclear cells (PBMCs) were extracted from the whole blood using Ficoll (Fisher Scientific, Waltham, USA) centrifugation. CD3^+^ T cells purified from PBMCs were activated using anti-CD3/28 beads at a 1:1 ratio and polarized into type 17 T cells with human IL-1β (20 ng/ml, BioLegend, San Diego, CA), IL-6 (20 ng/ml, BioLegend, San Diego, CA), and IL-23 (50 ng/ml, BioLegend, San Diego, CA). After five days, the cytokine levels in the supernatant were determined using ELISA (Multisciences, Hangzhou, China). The cells were collected for flow cytometry analysis.

### Ex vivo cytotoxicity assay

The EL4 cells were pulsed using 50 ng/ml OVA_257-264_ peptide (SIINFEKL) (Sangon Biotech, Shanghai, China) for 2 h at 37 °C and then labeled with 0.25 μM or 2.5 μM of CFSE (carboxyfluorescein succinimidyl ester; Thermo Fisher Scientific, Massachusetts, America) for 10 min at 37 °C. CFSE^l^°^w^ (SIINFEKL loaded target) and CFSE^high^ (irrelevant peptide control) EL4 cells were mixed at a 1:1 ratio and then co-cultured with Tc17 cells differentiated from OT-I T cells challenged (or not) with 8-074 at 30:1, 10:1, 3:1, and 1:1 effector to target cell ratios (E: T). The frequencies of the CFSE^l^°^w^ and CFSE^high^ EL4 cells in the CFSE positive fraction were determined using flow cytometric analysis 18 h after incubation, and the percent of the specific killing was calculated. Specific killing (%) = [1 − (Sample ratio) / (Negative control ratio)] × 100; Sample ratio = [CFSE^l^°^w^(target)/CFSE^high^(irrelevant)] value of each sample co-cultured with Tc17 cells; Negative control ratio = [CFSE^l^°^w^(target) / CFSE^high^ (irrelevant)] value of EL4 cells not cultured with Tc17 cells.

### Adoptive cell therapy tumor models

The B16-OVA tumor cells were implanted subcutaneously into the flank of C57BL/6 mice and allowed to grow. In parallel, splenocytes from OT-I mice were isolated and differentiated into Tc17 cells in vitro in the presence/absence of a 8-074 for five days. Once the tumor was measurable (normally between days seven and ten post-implant), the expanded T cells were injected intravenously. Antitumor responses were measured by assessing the tumor volume over time. The tumor volume was assessed once every two days using caliper measurement of the length and width of the tumor. The tumor volumes based on the caliper measurements were calculated using the modified ellipsoidal equation, where the tumor volume = 1/2 (length × width ^2^) [22]. Mice were euthanized after the tumor volume reached three ethical endpoints of 2,000 mm.

### Animal models

All experiments were approved by the IACUC and performed with strict adherence to a series of documents and standards of procedures (SOPs) relative to animal ethics and welfare. The mice were housed in cages with controlled temperature (25 ± 2 °C) and humidity (65 ± 5%) under a 12 h light/dark cycle. After a one-week adaptation period, six to eight-week-old female mice were injected s.c. with LLC (5 × 10^5^), B16F10 (2 × 10^5^), or MC38 (2 × 10^6^) cells into the lower right flank. Approximately seven days after the subcutaneous injection of tumor cells, the mice were randomly divided into four groups. RORγt agonist 8-074, LYC-55716 (BioChemPartner, Shanghai, China) and anti-PD-1 (BioXCell, New Hampshire, USA) treatment commenced when the average tumor size reached 50 mm^3^ for LLC and B16F10 and 150 mm^3^ for MC38. The 5 × 10^5^ LLC cells were transplanted subcutaneously into the right flank of the C57BL/6 mice seven days after being transplanted, and the mice were randomly divided into four groups. Anti-PD-1 was administrated one day after the RORγt agonist was treated (Clone: PMP1-14; 200 μg via intraperitoneal injection on day 1, 4, 7, 10, 13 after treatment with 8-074). For the CD8^+^ T cell depletion, mice were injected intraperitoneally (i.p.) with 400 μg of anti-CD8α (YTS 169.4; BioXCell, New Hampshire, USA) one day before and dosed per week after the anti-PD-1 treatment. Mice with established tumors were treated using intraperitoneal injection of 8-074 (indicated dose), LYC-55716 (50 mg/kg) or DMSO (SIGMA, New Jersey, USA) every day. Anti-PD-1 was dosed at 10 mg/kg every three days by intraperitoneal injection. The tumor volume based on the caliper measurements was calculated using the modified ellipsoidal equation, where the tumor volume = 1/2 (length × width ^2^) and the length was the longer dimension [22]. Two weeks after the 8-074 administration, the mice were sacrificed, and solid tumors were separated and photographed. TGI was calculated using the equation: [(C_t_ – C_0_) – (T_t_ – T_0_)] / (C_t_ – C_0_) × 100, where C_t_ = the mean tumor volume of the control group at the time (t); C_0_ = the mean tumor volume of the control group at t_0_; T_t_ = mean tumor volume of the treatment group at t; and T_0_ = mean tumor volume of the treatment group at t_0_.

### Tumor digestion

Tumors were harvested and cut into small pieces after removing connective tissue and tissue stroma. To obtain a single-cell tumor suspension, the small tumor pieces were incubated in an enzyme mixture of collagenase A (2 mg/ml, SIGMA, New Jersey, USA) and DNase-I (1 mg/ml, Roche, Basel, Switzerland) in an incomplete RPMI medium (Hyclone, Logan, Utah) for 30-60 min at 37 °C on a rocking platform. After digestion, the single-cell suspension was obtained by passing the digested tissue through a 40 μm nylon mesh. The resultant cells were washed twice in phosphate buffer solution (PBS) before staining for flow cytometry.

### FACS

Cells were stained with fluorochrome-labeled anti-mouse Ab such as CD45, CD3, CD4, CD8, Foxp3, IFN-γ, IL-17A, CD11b, CD11c, pAKT, pSTAT3, CCR6, or MHCII. For intracellular cytokine staining, single-cell suspensions from the tumor and TDLNs were stimulated using a cell stimulation cocktail (eBioscience, San Diego, California, USA, 500X used at 1X) consisting of PMA (40.5 μM, Cayman, Ann Arbor, Michigan, USA), ionomycin (670 μM, BioVision, San Francisco, USA), and protein transport inhibitors-brefeldin A (5.3 mM, Thermo, Massachusetts, America) and monensin (1 mM, Thermo, Massachusetts, America) for 6 h at 37 °C and 5% CO_2_. After 6 h, the cells were harvested and washed, surface stained with CD45, CD3, CD4, CD8, CD11b, CD11c, CCR6, and MHCII (FACS Buffer, Thermo, Massachusetts, America), fixed, permeabilized (IC fixation and Permeabilization buffer, Thermo, Massachusetts, America), and stained for pAKT, pSTAT3, IFN-γ, and IL-17A (Thermo, Massachusetts, America). Isotype controls with the same fluorochrome were used as controls. Cells were acquired using the FACS Aria II machine and analyzed using FlowJo software.

### Measurement of cytokines by ELISA and real-time PCR

The intracellular cytokines by TILs or in vitro differentiated T helper cells were quantified after restimulation with PMA plus ionomycin in the presence of GolgiStop for 6 h. The total RNA was isolated using the improved TRizol-based (Sigma, Darmstadt, Germany) method for qPCR analysis, and the mRNA expression was analyzed using a StepOnePlus (Life Technologies, Carlsbad, USA) real-time PCR instrument using housekeeping gene β-actin and Gapdh internal standards. qPCR was performed using A Power SYBR Green PCR Master Mix (Accurate Biology, Hunan, China) and two-cycle amplification for 40 cycles followed by the melting curve. The sequence of primers is listed in the Additional file 6: Table S1. In addition, the cytokines were quantified in cell-free culture supernatants using enzymelinked immunosorbent assay (ELISA) kit (the optical density (OD) value was measured at 450 nm, using 570 nm or 630 nm as the reference wavelengths, Multisciences, Hangzhou, China). The kit was used according to the manufacturer’s instructions [23].

### In vitro differentiation of the Mo-DC cells

Fluorescence staining panel for cell sorting of Mo-DC was assessed using flow cytometry. The Pan-DC (CD45^+^CD11c^+^) were enriched from C57BL/6 splenocyte lymphocytes with the EasySep™ Mouse Pan-DC Enrichment Kit from STEMCELL Technologies (Vancouver, Canada), then the cells were stained with CD45, CD11c, MHCII, CD11b, and Ly6c to obtain Mo-DC (CD45^+^CD11c^+^MHCII^+^CD11b^+^Ly6c^+^) through FACS.

### Transwell assays

Transwells with a 5-μm pore size (Costar, Corning, New York State, USA) were placed in a 24-well plate with 500 μl IMDM in the bottom chamber. 1) Different concentrations of recombinant murine CCL20 (PeproTech, Rocky Hill, USA) or 1 mg/ml neutralizing rat anti-CCL20 mAb (R&D Systems, Minn., USA) were added to the lower wells, and type 17 T cells were added to the upper wells. T cells were allowed to migrate through the Transwell membrane for 3 h at 37 °C. The migrated cells were then counted. 2) Different concentrations of recombinant murine CXCL10 (PeproTech, Rocky Hill, USA) or 1 mg/ml neutralizing rat anti-CXCL10 mAb (R&D Systems, Minn., USA) were added to the lower wells, and CD8^+^ T cells were added to the upper wells. The T cells were allowed to migrate through the Transwell membrane for 3 h and 6 h at 37 °C. The migrated cells were then counted. 3) Transwells with a 5 μm pore size (Costar, Corning, New York State, USA) were placed in a 24-well plate with 500 μL IMDM in the bottom chamber. 1 × 10^5^ sorted Mo-DC cells were added in the upper chamber. The lower chamber contained medium alone (-), or medium with different concentrations of recombinant CCL20 with/without neutralizing anti-(α) CCL20 mAb and neutralizing anti-CCR6 mAb. Plates were incubated for 6 h at 37 °C in 5% CO2, and the migrated Mo-DC were counted. 4) Different concentrations of cell culture supernatant of the Th17 cells after treatments or 1 mg/ml of neutralizing rat anti-CCL20 mAb were added to the lower wells, and the Mo-DC cells were added to the upper wells. The T cells were allowed to migrate through the Transwell membrane for 3 h and 6 h at 37 °C. The migrated cells were then counted.

### Pharmacokinetics

Male C57BL/6 mice were divided into two groups: 8-074 single intravenous injection group (2 mg/kg, *n* = 3) and 8-074 single gavage group (5 mg/kg, *n* = 3). After administration, blood samples were collected at 0.25, 0.5, 1, 2, 5, 7, and 24 h. Then the plasma samples were separated and stored at − 80 °C until the analysis. After being thawed at room temperature, 10 μL of plasma samples were added with 150 μL of precipitant containing the internal standard (verapamil 40 ng/mL) for the protein precipitation. The supernatant was mixed with a suitable volume of water and then analyzed using liquid chromatography in tandem with mass spectrometry (LC-MS/MS). The concentration of 8–074 in the plasma of the C57 mice after administration was determined using the inter-run standard curve samples (linear range of 3–10,000 ng/mL) and quality control samples.

### Biacore assay

Human nuclear receptor RORγt (residues 263–509)-GGG-SRC1 (SRC1 sequence: EKHKILHRLLQDS, Sangon Biotech, Shanghai). RORγt LBD was cloned in pET28a. Key residue mutations of RORγt LBD (such as PHE388, LEU391, CYS393, LEU396, ILE397, ILE400, CYS320, ALA321, LEU324, MET358, and PHE388) with LYC-55716 were cloned in pET28a. Key residue mutations of RORγt LBD (MET365, ALA368, PHE401, ILE400, ILE397, LEU396, TRP317, CYS320, LEU324, ALA327, TYR330, VAL331, MET358, and VAL361) with 8-074 were cloned in pET28a. The proteins were expressed in *E. coli* strain DE3. Then, the transformed *E. coli* culture was grown at 37 °C with 30 μg/mL Kanamycin LB (Luria–Bertani). When the OD_600_ of LB medium reached 0.6, the temperature was changed to 16 °C, and isopropyl-β-D-thiogalactopyranoside (IPTG, Beyotime, Shanghai, China) was added at a final concentration of 0.4 mM to induce protein expression for 16 h. After 16 h, the pellet was collected after centrifugation at 4,000 rpm for 15 min at 4 °C. RORγt protein was purified by Nickel Columns for Chromatography Nickel columns, and then the purified RORγt protein was concentrated in a 10 k enrichment tube (Millipore, Massachusetts, USA) and flash-frozen at − 80 °C. The RORγt LBD used in the binding assay was stored at − 80 °C in buffer containing 25 mM Hepes (Ph = 7.4), 200 mM NaCl, 5% glycerol.

The RORγt protein was immobilized on a CM5 chip (GE Health, Chicago, USA) using Biaocre 8 K. A sensogram was obtained using different serial concentrations of 8-074 (5000 nM, 2500 nM, 1250 nM, 625 nM, 312.5 nM, 156.25 nM, 78.125 nM, and 39.0625 nM). SPR sensorgrams have association time intervals of 40 s and dissociation time intervals of 60 s. Data were analyzed using Biacore Evaluation Software.

### RORγt dual FRET assay

The assay was performed according to a previous study [15, 24]. The plates were incubated for 1 h at room temperature and then read on Envision in LANCE mode configured for the europeum-APC labels.

### RORγt GAL4 cell-based reporter gene assay

The hRORγt LBD coding sequence was inserted into a pBIND expression vector (Promega, E1581) to express the ROR-GAL4 binding domain chimeric receptors. This expression vector and a reporter vector (pGL4.35, which carries a stably integrated GAL4 promoter-driven luciferase reporter gene [luc2P/9XGAL4 UAS/Hygro]) were co-transfected into the HEK293T host cells. The assay was performed according to a previous study [15]. EC_50_ of the sigmoidal fits were analyzed using Prism 5 and a four-parameter logistic fit equation, Y = bottom + (top–bottom) / (1 + 10(logEC_50_ − X) × hill slope). "X" is the log of compound concentration, and "Y" is the response, which increases as X increases. Y starts at "bottom" and goes to "top" with a sigmoid shape.

### Mouse Th17 differentiation assay

CD4^+^ T cells were purified from mouse splenocytes using a commercial CD4^+^ T cell negative selection kit (Invitrogen, California, USA). The 48-well plates were wrapped in the presence of anti-CD3 (0.25 mg/mL, Bioxcell, New Hampshire, USA) and anti-CD28 (1 mg/mL, Bioxcell, New Hampshire, USA) at 0 °C overnight. CD4^+^ T cells were skewed to Th17 cells by culturing cells in the presence of anti-IFNγ (10 mg/mL, Bioxcell, New Hampshire, USA), anti-IL-4 (10 mg/mL, Bioxcell, New Hampshire, USA), TGF-β (2 ng/mL, Peprotech, Rocky Hill, USA), and IL-6 (20 ng/mL, Peprotech, Rocky Hill, USA) for four days before analysis. Compounds or DMSO control were added to the culture on day 0 of Th17 differentiation at indicated concentrations. Percentage of IL-17 production from CD4^+^ T cells were analyzed by intracellular staining followed by flow cytometry. Dose–response curves were plotted to determine half-maximal inhibitory concentrations (EC_50_) for the compounds using the GraphPad Prism 5 (GraphPad Software, San Diego CA, USA).

### TCGA datasets

The Cancer Genome Atlas (TCGA) datasets were downloaded from cBioPortal (http://www.cbioportal.org/). According to gene median expression level, samples were divided into high and low expression groups. For RORC and PDCD1 expression analysis, we downloaded log2-transformed, normalized mRNA expression values (RSEM, Illumina HiSeq_RNASeqV2) and clinicopathological data TCGA cohort from the Cell Index Database CELLX. For the analysis of TCGA dataset (LUAD, BC, EAC, KIRC, and LIHC), a Kaplan–Meier curve was constructed to compare the overall and disease-free survival rates of the two groups. The log-rank *P* value and HR were calculated using SPSS 22.0. A correlation analysis of the gene expression in the tumor-infiltrating immune cells was analyzed using the Tumor Immune Estimation Resource (TIMER). SPSS 22.0 for windows (Chicago, IL, USA) was used for the data analysis, and statistical significance was determined using a t test. *P* values were then calculated. A *P* < 0.05 was considered statistically significant.

### Statistical analysis

In vitro experiments were done with biological replicates higher than or equal to three unless otherwise noted in the figure legends. Most critical experiments were conducted at least three times with similar results. Most data presented in the figures are mean ± SD of biological replicates. Statistics for in vitro data were done using Student t-test (two-tailed) by GraphPad Prism software. *P*-values < 0.0001, 0.001, 0.01 and 0.05 are represented as ****, ***, **and *, respectively.

## Results

### High RORC/IL-17A expression was associated with a good predictive value in human cancers

**Fig. 1:**
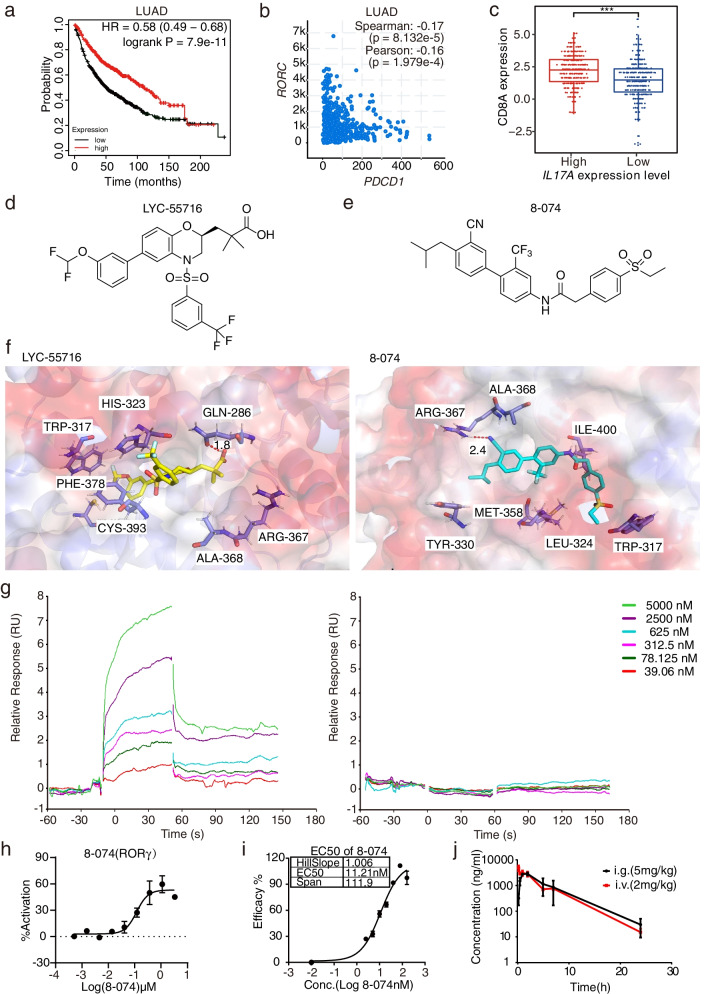
Identification of the selective RORγt agonist 8-074. **a** High expression of *RORC* correlated with a better prognosis in lung adenocarcinoma (LUAD) patients. Kaplan–Meier survival curves for patients with LUAG from TCGA. **b** The mRNA level of *PDCD1* was negatively correlated with *RORC* expression in LUAD in TCGA. **c** Expression of *CD8A* in the *IL17A* high expression and *IL17A* low expression groups in LUAD samples from TCGA (***P < 0.001). **d** and **e** Chemical structure of LYC-55716 or 8-074. **f** Key resides in RORγt protein (PDB ID: 6W9I; Resolution: 1.61 Å) binding to LYC-55716 (yellow stick) and 8-074 (green stick). The hydrogen bond interaction is shown as a red dotted line. Left: Biacore analysis of RORγt protein binding to LYC-55716. Right: Biacore analysis of RORγt protein binding to 8-074. **g** Biacore binding affinity determination. Sensorgram and saturation curve of the titration of 8-074 on the RORγt LBD protein or the RORγt LBD mutated protein. **h** The activity of 8-074 in Gal4 reporter gene assays of the three ROR subtypes. 8-074 showed RORγt agonist activity in the Gal4 reporter assay with an EC_50_ of 119 nM. **i** The dose–response curve was obtained by fitting a sigmoidal curve to data obtained from stimulation of mouse Tc17 cells using 8-074 concentrations ranging from 0.001 to 320 nM, Th17% was determined using flow cytometry. An EC_50_ value of 11.21 nM was obtained. **j** 8-074 (mg/kg) was injected into C57/BL6 mice via i.v. or i.g. The changes in the drug blood concentration in vivo were detected at different time points (*n* = 3). All error bars represent mean ± SD. Experiments were repeated three times with consistent results

To investigate the association of *RORC* expression with survival, we analyzed The Cancer Genome Atlas (TCGA) database. The high expression of *RORC* is associated with better survival in many types of cancer, such as breast carcinoma, esophageal adenocarcinoma, kidney renal clear cell carcinoma, hepatocellular liver carcinoma, and lung adenocarcinoma in TCGA (Fig. 1a, Fig. S1a). In all these cases, patients with high *RORC* expression had significantly higher survival rates than those with lower *RORC* expression. Furthermore, an exhausted T cell signature predicts a good response to immunotherapy [25]. To examine the correlation between the T cell exhaustion and RORγt signaling activation, we analyzed the correlation between the *RORC* expression and *PDCD1*, and this showed a negative correlation (Fig. 1b and Fig. S1b). In the analysis of the TCGA dataset, we found that the expression of *IL17A* was significantly positively correlated with the expression of *CD8A* in invasive breast carcinoma (BRCA), liver hepatocellular carcinoma (LIHC), and ovarian serous cystadenocarcinoma (OV) in lung adenocarcinoma in TCGA (Fig. S1c). In the analysis of the TCGA dataset, we also found that the expression of *IL17A* and *CD8A* was significantly positively correlated (*P* < 0.001) (Fig. 1c), and the tumor infiltration of CD8^+^ T cells was significantly higher in tumors with high expression of *IL17A* than that of *IL17A* with low expression (*P* < 0.001) (Fig. S1d). These results indicate that the high expression of *RORC* is correlated with better survival in multiple cancers, the high expression of *RORC* is inversely associated with the expression of co-inhibitory immune checkpoints, and high IL-17A is associated with high tumor infiltration of CD8^+^ T cells.

### Synthetic RORγt agonist enhanced Type 17 T cell differentiation and cytotoxic activity in vitro

To verify our hypothesis that RORγt agonist improves cancer immunity through the activation of Type 17 cells in the TME of lung cancer, we developed a potent and selective RORγt agonist 8-074 as a research tool based on our previous studies of the RORγt agonist [26]. To investigate the binding sites between the RORγt agonists (LYC-55716, 8-074) (Fig. 1d and e) and the RORγt protein, we took advantage of computational chemistry docking. We used a high-resolution crystal structure of the human RORγt protein (PDB ID: 6W9I; Resolution: 1.61 Å) and performed the computational docking of LYC-55716 and 8-074 with the standard precision (SP) mode of the Glide module (Fig. 1f, Fig. S1e). The carboxyl group of LYC-55716 acts as a hydrogen bond donor to form a hydrogen bond with GLN286, and the distance is 1.8 Å. The benzene ring connected to the trifluoromethyl group can form a π-π interaction with amino acid residue HIS323. In addition, LYC-55716 can also form hydrophobic interactions with multiple amino acid residues such as PHE388 and LEU391 (Fig. 1f, Fig. S1e). The docking results showed that an oxygen atom of the cyano group of 8-074 acts as a hydrogen bond acceptor to form a hydrogen bond with ARG367 at a distance of 2.4 Å. 8-074 could also form hydrophobic interactions with multiple amino acid residues such as MET365 and ALA368 (Fig. 1f, Fig. S1e). In the Biacore assay, 8-074 shows potent binding affinity to RORγt protein with a Kd at 497 nM but showed no binding affinity with the mutated RORγt protein at key binding sites (Fig. 1g). 8-074 not only displayed high selectivity versus the other two ROR members, but it also showed potent RORγt agonist activity in the Gal4 reporter gene assay with an EC_50_ value of 118.7 nM (Fig. 1h, Fig. S2a-b). 8-074 showed EC_50_ in the dual FRET was 19.95 nM, while LYC-55716 in the dual FRET was EC_50_ = 30 nM [18]. 8-074 demonstrates stimulating the mouse Tc17 cells with EC_50_ at 11.21 nM (Fig. 1i). Collectively, 8-074 demonstrated potent and selective binding to the RORγt protein as well as potent in vitro activity compared with LYC-55716.

Pharmacokinetic experiments were performed in the C57 mice (Fig. 1j and Additional file 7: Table S2). After a single intragastric administration of 5 mg/kg of 8-074, the half-life of 8-074 was 3.34 ± 0.52 h, and the clearance rate was 38.3 ± 18.1 mL/min/kg. This result indicated a favorable metabolic property. In addition, 8-074 showed a good bioavailability of 38% and half-life characteristics, indicating that daily administration is feasible. The plasma AUC value of 8-074 is 20600 ± 1500 h·ng/mL (Fig. 1j and Additional file 7: Table S2). The low clearance rate and long half-life of 8-074 in mice indicated that sustained anti-tumor effects may be obtained in humans. In summary, 8-074 showed favorable pharmacokinetic characteristics in vivo.

**Fig. 2:**
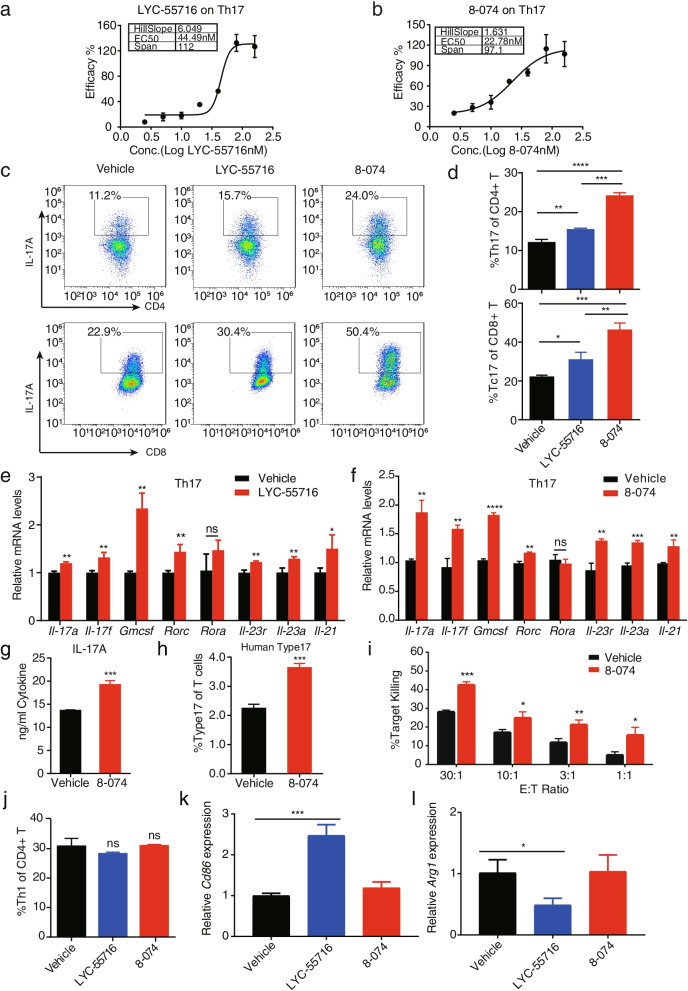
RORγt agonists enhance Type 17 T cell differentiation and cytokine production. **a** and **b** The dose–response curve of the stimulation of mouse Th17 cells using LYC-55716 or 8-074 concentrations ranging from 0 to 160 nM. The IL-17A concentration was determined using ELISA. **c** Representative flow graph. RORγt agonists increased the percentage of CD4^+^ IL-17A^+^ (Th17, upper) and CD8^+^ IL-17A^+^ (Tc17, bottom) cells. The extent of Th17 and Tc17 cell differentiation was then assessed using intracellular staining. **d** Statistical results of **c** (*P < 0.05, **P < 0.01, ***P < 0.001, ****P < 0.0001). **e** and **f** qPCR analysis of the Th17 signature cytokines expression of the Th17 cells after LYC-55716 or 8-074 treatment, *P < 0.05, **P < 0.01, ***P < 0.001, ****P < 0.000. **g** IL-17A levels in 8-074 treated Type 17 T cells. The IL-17A levels were assayed by ELISA (***P < 0.001). **h** The percentages of the Type 17 T cells in the CD3^+^ T cells were detected by flow cytometry. The human total CD3^+^ T were differentiated under Th17 polarization conditions for five days. (***P < 0.001). **i** The cytotoxic activity of the 8-074-treated Tc17 cells differentiated from the OT-I T cells against OVA-pulsed EL-4 lymphoma cells in vitro***.*** (*P < 0.05; **P < 0.01; ***P < 0.001). **j** Representative flow graph of Th1 cell in CD4^+^ T. **k** mRNA expression of the *Cd86* levels of B cells as determined by a qPCR assay. *** *P* < 0.001. **l** mRNA expression of the *Arg1* levels of macrophages as determined by a qPCR assay. *P < 0.05. The Student’s test was used for the statistical test. All error bars represent mean ± SD. Experiments were repeated three times with consistent results

To compare the effect of LYC-55716 and 8-074 on the promotion of secretion of IL-17A by Th17 cells, we used the ELISA to measure the supernatant of Th17 cells. The EC_50_ of LYC-55716 and 8-074 in IL-17A secretion in Th17 cells were 44.49 nM and 22.78 nM, respectively (Fig. 2a-b). To determine whether a synthetic compound could modulate RORγt activity, we tested the effects of LYC-55716 and 8-074 on murine Th17 and Tc17 differentiation. 8-074 significantly increased the percentage of CD4^+^ T and CD8^+^ T cells that express IL-17A (from 12.1% to 24.0% in CD4^+^ T and 22.9% to 50.4% in CD8^+^ T cells) superior to that of LYC-55716 at the same concentration (Fig. 2c-d). Collectively, 8-074 showed improved potency to promote IL-17A secretion and Type 17 differentiation than LYC-55716.

To measure the signature cytokine expression change of Type 17 cells after 8-074 and LYC-55716 treatment, the related cytokines from the treated Th17 cells were analyzed by qPCR (Fig. 2e-f). Both LYC-55716 and 8-074 upregulated the signature cytokine mRNA expression of the Th17 cell and promoted transcription factor RORγt but not RORα (Fig. 2e-f). The production of IL-17A, IL-17F, and IL-22 was increased by 8-074 treated CD3^+^ T cells polarized under Type 17 T cell conditions compared with vehicle alone (Fig. 2g, Fig. S2c). 8-074 also had a similar effect of promoting Type 17 cell differentiation on primary human T cells. Flow cytometry results showed that 8-074 enhanced Type 17 T cell differentiation compared with vehicle-treated Type 17 T cells (Fig. 2h). In summary, 8-074 was demonstrated to promote cytokine secretion in Th17 cells.

In a recent study, the RORγ agonist LYC-54143 was found to enhance the direct tumor-killing activity of Tc17 cells in vitro and showed robust tumor growth inhibition in tumor-bearing mice [9]. As shown in Fig. 2i, the 8-074-treated Tc17 cells exhibited a significant increase in cytotoxic killing activity. To exclude the direct cytotoxic killing effect of 8-074 on tumor cells, we conducted an apoptosis assay, a cell proliferation assay, and a cytotoxic toxicity assay on tumor cells upon 8-074 treatment. The results showed that, even at higher concentrations of 8-074, apoptosis was not observed in the tumor EL4 cells (Fig. S2d), and cytotoxicity was not observed either (Fig. S2e-f), indicating that 8-074 had no direct toxic effect on tumor cells. Thus, our data collectively suggested that the in vitro treatment of T cells with the RORγt agonist 8-074 enhanced Tc17 cytotoxic function directly.

To confirm the in vitro specificity of 8-074, splenocytes from C57BL/6 wild-type mice were activated by plate-bound anti-CD3 and anti-CD28 antibodies and polarized with anti-IL-4 and IL-12 to obtain Th1 cells. IFN-γ, a signature cytokine of Th1 cells, was not affected by LYC-55716 or 8-074 compared with vehicle alone (Fig. 2j, Fig. S2g). There was no significant change in gene expression of the activated B cell markers *Cd19* and *Cd86* in the 8-074 treatment group compared with the vehicle, but there was a significant increase in the *Cd86* gene expression in the LYC-55716 treatment group compared with vehicle (Fig. 2k, Fig. S2h). There was no significant change in the gene expression of macrophage markers *Arg1*, *Il-1β*, *Il-6,* and *Il-10* in the 8-074 treatment group compared with vehicle, but there was a significant decrease in *Arg1*, *Il-1β*, *Il-6,* and *Il-10* gene expression in the LYC-55716 treatment group compared with vehicle (Fig. 2l, Fig. S2i-k). 8–074 showed better potency in multiple in vitro assays and better selectivity in macrophage and active B cells than LYC-55716. Hence, we chose to primarily use 8-074 as a tool to investigate the mechanism of action of RORγt agonism in TME in vivo.

### RORγt agonist showed robust antitumor efficacy in syngeneic tumor models, and 8-074 improved the efficacy of anti‑PD‑1 therapy in a murine lung cancer model

**Fig. 3:**
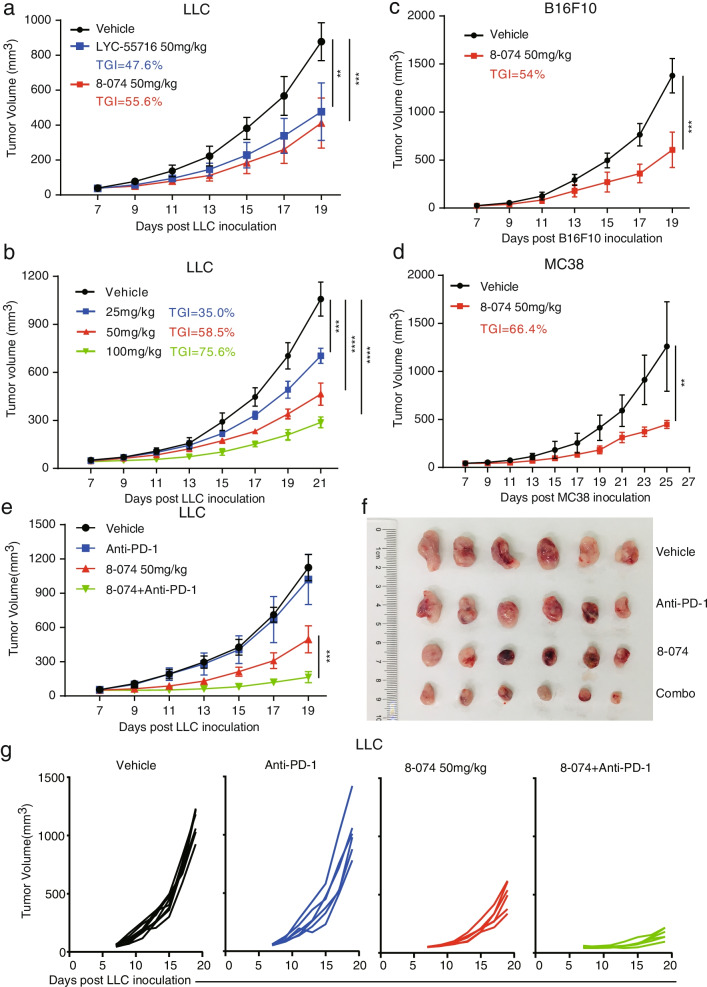
RORγt agonist 8-074 inhibits tumor growth in syngeneic models and improves anti‐PD‐1 therapy in LLC. **a** C57BL/6 inoculated with LLC cells were treated with a vehicle control, 8-074, or LYC-55716 at 50 mg/kg via i.p. injection, QD over two weeks (*N* = 5–8 per group, **P < 0.01 and ***P < 0.001). **b** Dose-escalation study of 8-074 treatment in LLC model. C57BL/6 mice bearing LLC tumors were randomly divided into four groups (*n* = 5–8 per group) and administered with 8-074 (0, 25, 50, 100 mg/kg) via i.p. injection, QD over two weeks. ***P < 0.001 and ****P < 0.0001, by 2-way ANOVA. **c** Mice were injected subcutaneously with 2 × 10^5^ B16F10 cells or **d** 2 × 10^6^ MC38 cells on C57BL/6 mice treated with RORγt agonist 8–074 via intraperitoneal injection, QD over two weeks. *N* = 5–8 per group. **P < 0.01 and ***P < 0.001. **e** Combination treatment of 8-074 and anti-PD-1 showed significant tumor reduction in the LLC model. *N* = 6 mice per group. ***P < 0.001, by 2-way ANOVA. **f** The images of tumors of mice in four groups were obtained from sacrificed mice at the end of this experiment. **g** Combination treatment of 8-074 and anti-PD-1 showed significant tumor reduction in the LLC model. *N* = 6 mice per group. Tumor growth curve of individual mice of the IgG group, anti-PD-1 group, 8-074 group, and the combination of anti-PD-1 antibody with 8-074 group. Tumor volume is represented as the mean ± SD. All error bars represent mean ± SD. Experiments were repeated three times with consistent results

We further explored the in vivo efficacy of 8-074. First, we conducted a drug toxicity evaluation by histopathology using H&E staining. No noteworthy necrosis or other abnormality was observed, demonstrating that the 8-074 itself had some acute toxic effects on these organs but did not cause a severe inflammatory response (Fig. S3a). 8-074 showed better antitumor activity than LYC-55716 at the same dose in our LLC model (Tumor growth inhibition (TGI): 55.6% vs. 47.6%; Fig. 3a). The body weight changes are shown in the Fig. S3b. Our results showed that an obvious TGI (Fig. 3b) and a slight body weight loss in mice (Fig. S3c) had occurred in our LLC model after the 8-074 treatment. Given the in vivo toxicity and body weight loss during dose escalation, we determined 50 mg/kg for use in the in vivo mechanistic study. The anti-tumor effect of 8-074 was also tested in two other murine syngeneic tumor models (B16F10 and MC38, which are models of melanoma and colon adenocarcinoma, respectively). Tumor growth was significantly inhibited in mice treated with 8-074 compared to mice that received vehicle (Fig. 3c-d). In addition, we found that the combination of anti-PD-1 and 8-074 led to a more pronounced inhibitory effect on tumor growth than 8-074 or anti-PD-1 alone (Fig. 3e-g). It was found that the tumor growth rate of mice in the anti-PD-1 + 8-074 group (TGI: 89.7%) was significantly slower than 8-074 alone (TGI: 59.3%) and anti-PD-1 alone (TGI: 12%) (Fig. 3e). In conclusion, 8-074 exhibited a significant antitumor efficiency in various syngeneic tumor models and was synergistic with anti-PD-1.

### RORγt agonist treatment resulted in increased intratumoral CD8^+^ T cell numbers

**Fig. 4:**
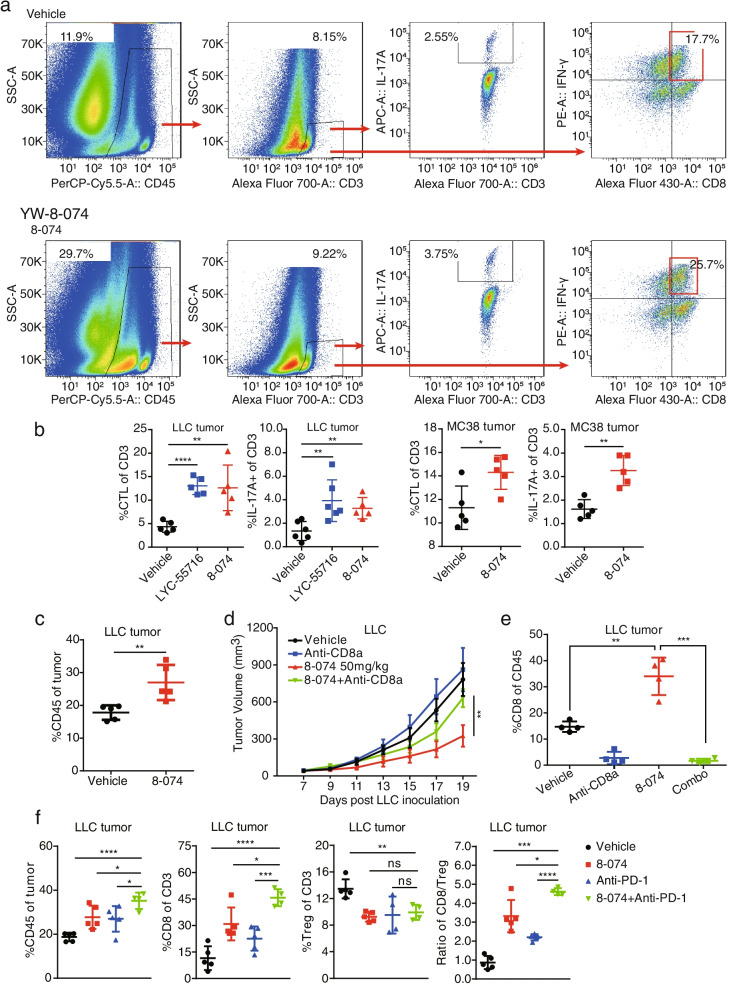
RORγt agonist 8-074 promotes CD8^+^ T and reduces Treg tumor infiltration in the LLC model. **a** Flow cytometry analysis of TIICs from LLC tumor-bearing mice in Fig. 3a, vehicle group and 8-074 (50 mg/kg) group. Mice with established LLC tumors were treated with vehicle or 8-074 (50 mg/kg) QD for 14 days. The percentage of CD45^+^ cells in the tumor was shown. The proportion of IL-17A^+^ and CTL cells among the CD3^+^ cell populations is also shown. **b** Statistical results of Flow Cytometry (FCM) analysis for CTL and IL-17A^+^ cells among CD3^+^ population in LLC and MC38 tumor. *P < 0.05, **P < 0.01, and ****P < 0.0001. **c** Statistical results of the FCM analysis for the CD45^+^ cells in the LLC tumor population. A student’s t-test was used for the statistical test (*N* = 5 per group, **P < 0.01). **d** Cumulative graph of the mean tumor size per group after CD8^+^ T cell depletion. **P < 0.01 by 2-way ANOVA. For CD8^+^ T cell depletion, mice were injected i.p. with 400 μg of anti-CD8α (YTS 169.4; BioXCell) one day before and dose per week after anti-PD-1 treatment. **e** CD8^+^ T cell populations of tumors from mice each group (**P < 0.01, and ***P < 0.001). **f** The percentage of CD45^+^ cells in the tumor and the proportion of CD8^+^ T and Treg among the CD3^+^ populations are shown. The Student’s t-test was used for the statistical test. Data represent mean ± SD of biological quadruplicates. Experiments were repeated three times with consistent results

Growing evidence supports the notion that CD8^+^ T cells in the TME are associated with clinical response to anti-PD-1 [19, 27]. We thus analyzed the CD8^+^ T cell infiltration in TME in RORγt agonist-treated tumors. Flow cytometry shows that treatment with RORγt agonist led to a marked increase of Type 17 T cells (IL-17A^+^) and CTL cells (CD8^+^ IFN-γ^+^) in the CD3^+^ T cell (Fig. 4a-b). Compared with the vehicle group, the proportion of total immune cells (CD45^+^) in the LLC tumor tissues in the 8-074 treatment group increased (*P* < 0.01) (Fig. 4c). To investigate the contribution of CD8^+^ T cells to the anti-tumor activity of 8-074 directly, CD8^+^ T cells were depleted in the LLC model using anti-CD8α. CD8^+^ T cell depletion reduced the overall efficacy of the tumor inhibition mediated by 8-074 to a large extent. The CD8^+^ T depletion in tumors was confirmed by a flow cytometry analysis (Fig. 4d-e). We also analyzed the infiltration of immune cells in the tumor tissues of each group of mice, as shown in Fig. 4f. We found that the combination of 8-074 and anti-PD-1 increased the total tumor immune cell infiltration and CD8^+^ T cell infiltration in the LLC mice compared with 8-074 alone and anti-PD-1 alone. However, compared with anti-PD-1 alone, the Treg cells in the tumor of the combination group had a tendency to decrease, while the CD8^+^ T/Treg ratio increased significantly (Fig. 4f). In addition, a noteworthy decrease of Treg and a remarkable elevation of CD8^+^ T/Treg ratio were also observed in the 8-074-treated LLC tumors (Fig. S4a-b). These findings demonstrated that 8-074 promoted CD8^+^ T cell tumor infiltration, reduced Treg tumor infiltration and the combination of 8-074 and anti-PD-1 induced a pharmacologically superimposed or synergistic effect. Collectively, these results demonstrated that the RORγt agonist treatment modulated TME and promoted CTL tumor infiltration favoring the immune reaction.

To investigate the potential immune pathway involved, we performed quantitative PCR (qPCR) to detect the expression level of *Ifn-γ* in the tumors of LLC mice. The expression level of *Ifn-γ* in the tumors in the 8-074 group was increased (Fig. S4c). An ELISA experiment also verified the increase of the secreted IFN-γ protein in the tumor tissues of mice treated with 8-074 in LLC model mice (Fig. S4d), suggesting that the expressions of IL-17A and IFN-γ are positively correlated.

### RORγt agonist-dependent upregulation of CD8^+^ T infiltration was correlated with CXCL10 and Mo-DC

**Fig. 5:**
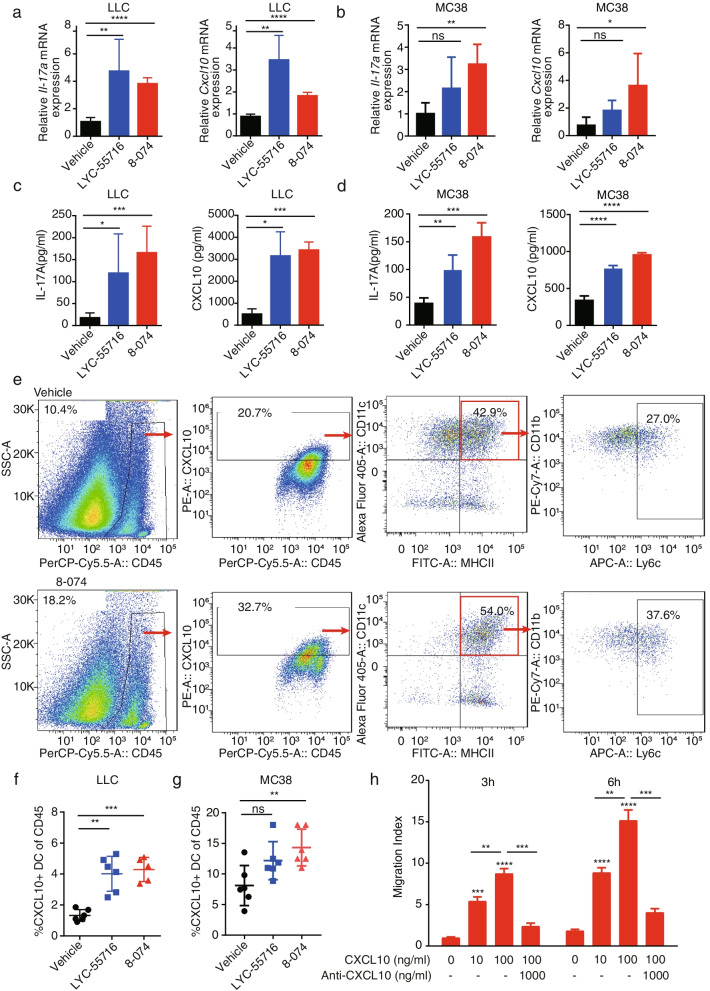
Tumor infiltration of CD8^+^ T cells mediated by RORγt agonist 8-074 is correlated with CXCL10^+^ DC. **a** and **b** The relative mRNA expression of *Il-17a* and *Cxcl10* levels of the LLC tumor (**a**) and the MC38 tumor (**b**) as determined by a qPCR assay (*P < 0.05, **P < 0.01, ****P < 0.0001). **c** and **d** ELISA assayed the relative cytokine level of IL-17A and CXCL10 in the LLC tumors (**c**) and the MC38 tumor (**d**). *P < 0.05, **P < 0.01, ***P < 0.001, ****P < 0.0001. **e** Representative flow panels for an analysis of CXCL10^+^ DC in the LLC tumor. **f** and **g** Infiltration of CXCL10^+^ DC in the LLC tumor (**f**) and the MC38 tumor (**g**) Statistical result of FCM analysis for CD11c^+^ MHCII^+^ CD11b^+^ Ly6c^+^ CXCL10^+^ DC among CD45^+^ cells in tumors (**P < 0.01, ***P < 0.001). **h** Migration of CD8^+^ T cell is mediated by CXCL10. Migration in the Transwell experiments of CD8^+^ T cells in the presence of different concentrations of CXCL10 in the presence or absence of blocking anti-CXCL10 mAb for 3 h or 6 h. *N* = 3, ** *P* < 0.01, *** *P* < 0.001 and **** *P* < 0.0001. The Student’s t-test was used for the statistical test. Data represent mean ± SD of biological quadruplicates. Experiments were repeated three times with consistent results

To further explore the molecular mechanism mediated by 8-074, we looked for clues from the chemokine promoted by 8-074 treatment. Recent evidence suggests that the dominant chemokines for recruitment of effector CD8^+^ T cells are those that engage the chemokine receptor CXCR3 [28]. Furthermore, expression of the CXCR3-cognate chemokines (CXCL9 and CXCL10) is correlated with T cell infiltration status [29, 30]. For validation, we detected the expression of *Il-17a* and *Cxcl10* levels in LLC and MC38 mice tumors, respectively, using qPCR (Fig. 5a-b) and ELISA (Fig. 5c-d). The expression levels of *Il-17a* in RORγt agonist-treated mice tumors were increased, and *Cxcl10* was also significantly upregulated. These data suggested a substantial correlation between IL-17A and the CD8^+^ T cell infiltration in LLC tumors and indicated a probable mechanism of the indirect anti-tumor effect of Type 17 T cells.

CXCL10 expression is induced upon Type I IFN production by antigen-presenting cells (APCs) and facilitated CD8^+^ cytotoxic T cells [31]. Although IFN-γ induced CXCL10 is secreted by various cell types, the major source of CXCL10 at the tumor site is CD11b^+^ myeloid cells [32, 33]. To further explore the correlation between CXCL10 and RORγt agonist, we analyzed the CXCL10 secreting monocyte-derived dendritic cells (MoDCs) in LLC (Fig. 5e) and MC38 (Fig. S5a) tumors after RORγt agonist treatment by flow cytometry. We found that treatment of RORγt agonist led to a marked increase of intratumoral CXCL10^+^ DCs among the CD45^+^ populations (Fig. 5f-g). Then we verified the effect of CXCL10 on CD8^+^ T cell migration using a Transwell experiment. Conversely, when neutralizing chemokine CXCL10 in the Transwell with anti-CXCL10, the migration of CD8^+^ T cells was significantly reduced (Fig. 5h). Our results indicated that the RORγt agonist promoted CD8^+^ T cell tumor infiltration by increasing MoDCs, which secrete CXCL10 in the TME.

**Fig. 6:**
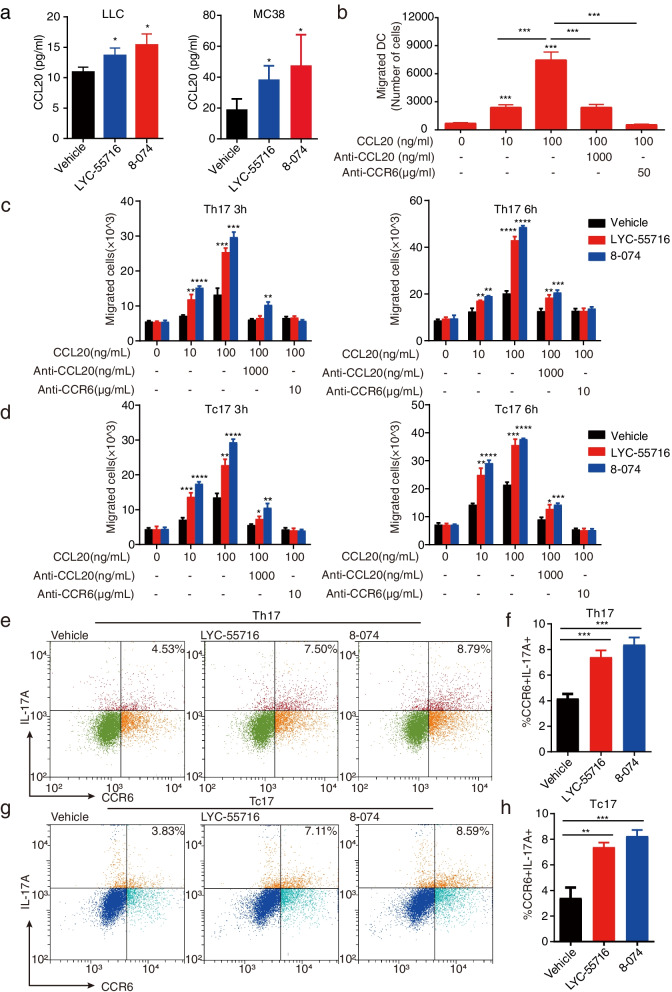
RORγt agonist promotes CCL20-CCR6 signaling and enhances Type 17 T cell migration. **a** Relative cytokine level of CCL20 in the LLC tumors and the MC38 tumor from the vehicle or 8-074 treated ELISA analyzed mice (*P < 0.05). **b** Transwell assay of the CCL20-CCR6 mediated Mo-DC migration. Statistical result of the migrated cells in the bottom chamber was shown (*N* = 3, ***P < 0.001). **c** and **d** Validation of the CCL20-CCR6 mediated Transwell migration assay. 1 × 10^5^ Th17 cells (**c**) or Tc17 cells (**d**) were added to the upper chamber. The lower chamber contained medium alone (-), or medium with recombinant CCL20, neutralizing anti-(α) CCL20 mAb, neutralizing anti-CCR6 mAb. The migrated cells in the lower chamber were counted after 3 h or 6 h. (*P < 0.05; **P < 0.01; ***P < 0.001 and ****P < 0.001). **e** Representative flow cytometry graph for the analysis of CCR6 in Th17 cells. **f** Statistical results of FCM analysis for CCR6^+^ IL-17A^+^ cells among the CD4^+^ T cells in Fig. 6e. A Student’s t-test was used for determining significance (***P < 0.001). **g** Representative flow panels of the analysis of CCR6 in Tc17 cells. **h** Statistical results of FCM analysis for CCR6^+^ IL-17A^+^ cells among the CD4^+^ T cells in Fig. 6g. A Student’s t-test was used for determining significance (**P < 0.01, ***P < 0.001). The student’s t-test was used for the statistical test. Data represent mean ± SD of biological quadruplicates. Experiments were repeated three times with consistent results

Most MoDCs express CCR6, which plays an important role in controlling the trafficking of DC via the CCR6-CCL20 axis [21, 34]. In addition, CCL20 functions in the recruitment of inflammatory cells by binding to CCR6 expressed on DCs, neutrophils, and memory T lymphocytes [35, 36]. CCR6 has also been reported to express on MoDC cells [37]. Consistently, LLC and MC38 tumor-infiltrating CD11c^+^ MHCII^+^ CD11b^+^ Ly6c^+^ CXCL10^+^ DCs were significantly increased in CD45^+^ cells in the RORγt agonist-treated group compared with the vehicle group, and CCL20 levels were higher in RORγt agonist-treated tumor tissue compared to vehicle treated tumors (Fig. 6a). Moreover, in an in vitro migration assay using MoDCs, results demonstrated that CCL20 induced Mo-DC migration in a CCL20 and CCR6 dependent manner (Fig. 6b). These results suggested that the CCL20-CCR6 axis may play an important role in inducing Mo-DC migration.

### RORγt agonist promoted Type 17 T cell migration by upregulating CCL20 and CCR6 expression

Type 17 T cells can express the chemokine receptor CCR6, which guides cells to regions of high CCL20 [38]. We further conducted a Transwell migration to measure the Type 17 cell migrations towards CCL20. After five days of differentiation, Th17 and Tc17 cells were harvested and assayed for chemotaxis using Transwell units. The CCR6-CCL20 axis mediated chemotaxis is showed in dose-dependent and time-dependent manners (Fig. 6c-d). A recent study also reported that RORγ agonists increased the expression of CCR6 and CCL20 in Tc17 cells, and this might contribute to the better migration of Tc17 cells into the tumor and recruitment of Tc1 cells [9]. Thus, we further tested the effect of 8-074 on the expression of CCR6 during the differentiation of Type 17 T cells. On the fifth day of differentiation after 8-074 treatment, the ratio of IL-17A^+^ and CCR6^+^ cells in CD4^+^ T cells and CD8^+^ T cells in the 8-074 treatment group increased significantly (Fig. 6e-h). In summary, these data showed that the RORγt agonist increased *CCR6* expression, and high concentrations of CCL20 attracted CCR6-expressing immune cells such as Type 17 cells.

### RORγt agonist promoted Mo-DC cell migration via CCL20 secreted from Th17/Tc17

**Fig. 7:**
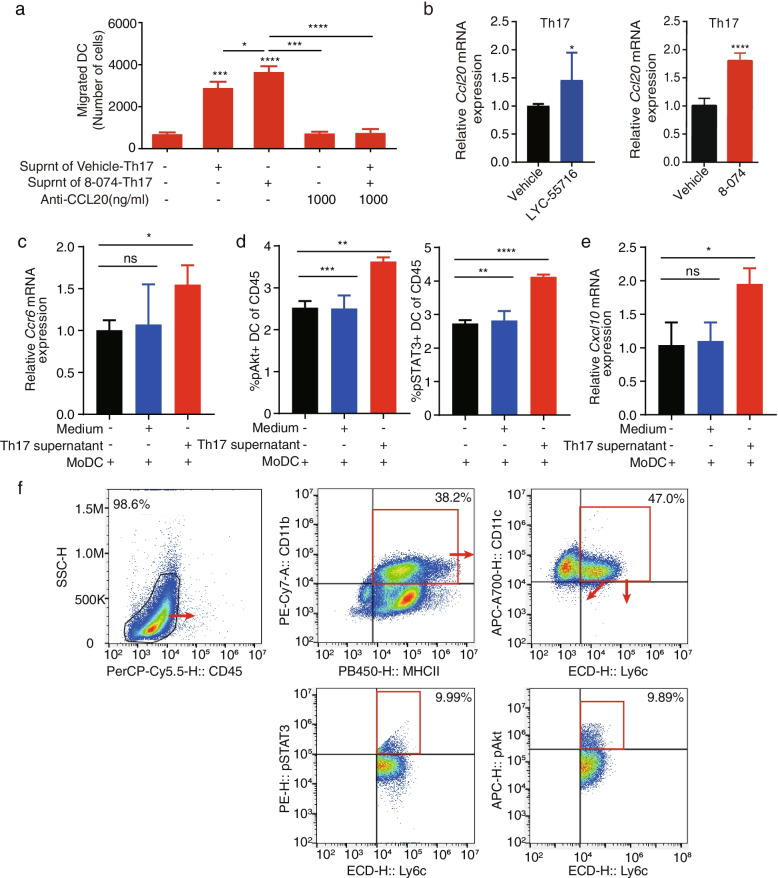
Type17 T cell activates the Ccr6-pAKT-STAT3-Cxcl10 axis in the MoDC. **a** Statistical results on the migrated Mo-DCs in the bottom chamber. Data shown is the mean ± SD from a representative experiment. *N* = 3, * *P* < 0.05; *** *P* < 0.001 and **** *P* < 0.0001 by Student’s t-test. **b** mRNA level of *Ccl20* in Th17 cells with or without LYC-55716 or 8-074 by qPCR, respectively (*P < 0.05, ****P < 0.0001). **c** The mRNA expression level of *Ccr6* in the DC cells treated with the Th17 supernatant was detected by qPCR compared with the medium only (*P < 0.05). **d** The ratio of pAKT^+^ DC cells and pSTAT3^+^ DC cells to the CD45^+^ cells in the MoDC treated with the Th17 supernatant was measured by flow cytometry compared with the MoDC treated with the medium only (**P < 0.01, ****P < 0.0001). **e** The expression level of *Cxcl10* mRNA in the MoDC treated with the Th17 supernatant was detected by qPCR compared with the MoDC treated with the medium only (*P < 0.05). **f** Representative flow cytometry plots for the analysis of the pAKT^+^ DC cells and pSTAT3^+^ DC cells in the CD45^+^ cells. Data are shown as the mean ± SD of a representative experiment, and Student’s t-test was used for the statistical test. Experiments were repeated three times with consistent results

It was reported that CCL20 in a tumor could recruit Th17 in TIME [39]. We found that the RORγt agonist 8-074 upregulated the expression of the chemokine CCL20, which mediated the migration of Mo-DCs (Fig. 6a-b). We then sought to identify the connections between CCL20 and RORγt as well as between CCL20 and Type 17 T cells (Fig. 7a). We collected the supernatant of Th17 cell after the 8-074 treatment to verify the effect on Mo-DC migration. Our results showed that the Th17 cell supernatant promoted the migration of Mo-DC (Fig. 7a). When anti-CCL20 was added to the supernatant of Th17 cells, the induced Mo-DC migration effect was neutralized (Fig. 7a). Taken together, these data suggested that the RORγt agonist promoted the migration of Type 17 T cells with enhanced CCL20 production; and CCL20 mediated the migration of Mo-DC.

In Fig. 5a and c, we show that an increase in IL-17A content in the tumor tissues of LLC mice treated with 8-074 was observed compared with vehicle alone. We also tested whether the increase in *Ccl20* in Th17/Tc17 was also induced by the 8-074 treatment. As expected, the RORγt agonist LYC-55716 and 8-074 promoted the expression of *Ccl20* in Th17/Tc17 cells (Fig. 7b, Fig. S5b-c). These data suggested that 8-074 promoted Th17/Tc17 cells to secrete CCL20 and then may have enhanced the Mo-DC tumor infiltration mediated by the CCL20-CCR6 axis.

Next, we further explored how Th17 cells affected the CXCL10^+^ MoDCs. We found that 8-074 promoted the Th17 cells to secrete CCL20 (Fig. 7b, Fig. S5c). Because most Mo-DCs express CCR6, we hypothesized that CCR6 plays an important role in controlling DC migration through the CCR6-CCL20 axis [21, 34]. Therefore, we found through qPCR experiments that, compared with the medium alone, the expression of *Ccr6* in the DC cells was significantly increased in the supernatant of the culture medium from the Th17 cells (Fig. 7c). We also found by flow cytometry that the proportion of pAKT^+^ Mo-DC cells and pSTAT3^+^ Mo-DC cells in the CD45^+^ cells was significantly up-regulated after the supernatant of the cultured Th17 cells was treated with the DC cells (Fig. 7d and f). The expression of *Cxcl10* was significantly increased when the DC cells were treated with the culture supernatant of the Th17 cells compared with the medium alone (Fig. 7e). In summary, we concluded that Th17 secreted CCL20 that bound to CCR6 on Mo-DC and then promoted the phosphorylation of AKT and STAT3 to activate the secretion of CXCL10 in the MoDC.

Collectively, 8–074 can promote the differentiation of Type 17 T cells and enhance the function of the cell itself, namely the secretion of cytokines, such as IL-17A and CCL20, and the expression of cell surface chemokine receptors such as CCR6. CCL20 secreted by the Th17 cells binds to CCR6 on the surface of the MoDC to promote the phosphorylation of AKT and STAT3, which in turn promotes the expression of the CXCL10^+^ MoDCs.

### RORγt agonist 8-074-treated Tc17 cells had an improved migration capability in vivo and recruited CD8^+^ T cells to the TME

**Fig. 8:**
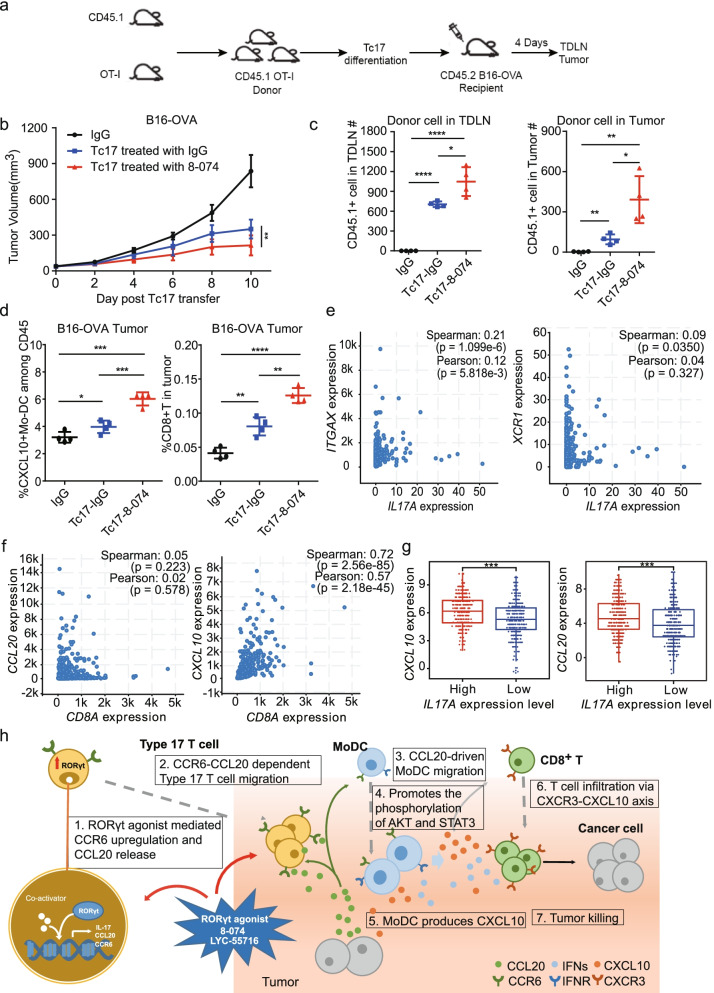
RORγt agonist treated Tc17 cells enhanced tumor CD8^+^ T cell infiltration in mice and IL17A is associated with immune cell infiltration in lung adenocarcinoma. **a** Schematic diagram of the experimental scheme. **b** ADT of RORγt agonist 8-074 treated Tc17 cells showed superior TGI in mice implanted with B16-OVA tumor cells. (*N* = 4 per group). **P < 0.01, by 2-way ANOVA. **c** RORγt agonist 8-074 enhances Tc17 cell migration in vivo. Two days after the last transfer into B16-OVA tumor-bearing mice, the number of CD45.1^+^ donor cells in the TDLN and tumor were analyzed by flow cytometry (*P < 0.05; **P < 0.01 and **** *P* < 0.0001). **d** RORγt agonist 8-074 increases tumor infiltration of CXCL10^+^ DC and CD8^+^ T cells (*P < 0.05; **P < 0.01 and ****P < 0.0001). **e** Left: *IL17A* expression correlationship with *ITGAX* expression; Right: *IL17A* expression correlationship with *XCR1* expression in LUAD in the TCGA. **f** Left: *CCL20* expression correlationship with *CD8A* expression; Right: *CXCL10* and *CD8A* expression correlationship in LUAD samples from the TCGA. **g** Expression of *CXCL10* and *CCL20* in *IL17A* high expression and low expression groups in LUAD in the TCGA (***P < 0.001). **h** Schematic figure illustrating the mechanism of type 17 T cells modulating the TME. Data are shown as the mean ± SD of a representative experiment, and a student’s t-test was used for the statistical test. Experiments were repeated three times with consistent results

To further verify the above mechanism, we used an adoptive transfer assay (ADT) to import Tc17 cells into the tumor mouse model to observe the changes in the TME (Fig. 8a). Equal numbers of Tc17 cells differentiated in vitro with or without RORγt agonist were injected into B16-OVA tumor-bearing mice by tail vein injection. We observed tumor shrinkage with Tc17 cells, and 8-074-treated Tc17 cells showed superior antitumor activity (Fig. 8b), thus confirming our in vitro findings (Fig. 2i).

We hypothesized that Tc17 cells may migrate to the tumor tissue, secrete CCL20 to recruit MoDCs, and then the activated DCs secrete CXCL10 to induce CD8^+^ T cell tumor infiltration, leading to inhibition of tumor growth. To verify our hypothesis, we analyzed the changes of immune cell components in tumor-draining lymph node (TDLN) and the tumor tissue of mice after the ADT experiment. Tc17 can migrate to TDLN and tumors after being injected into tumor-bearing mice (Fig. 8c). More donor cells (CD45.1^+^) were detected in lymph nodes and tumors of mice, which were injected with 8-074 treated Tc17 cells, compared with controls, indicating that 8-074 can enhance the migration ability of Tc17 cells in vivo. Furthermore, the input of Tc17 cells enhanced the infiltration of CXCL10^+^ MoDCs and CD8^+^ T cells in the tumor tissues of each group (Fig. 8d), suggesting that the antitumor effect of Tc17 in vivo is not only from a direct cytotoxic killing effect of Tc17, but also from promoting the recruitment of CD8^+^ T cells into tumors. We also found that the number of CXCL10^+^ MoDCs and CD8^+^ T cells in tumors of mice injected with 8–074 treated Tc17 cells was higher than in the tumors of mice injected with vehicle-treated Tc17 cells (Fig. 8d).

Together, our results imply that Type17 cells secrete CCL20 to mediate DC migration to the tumor, resulting in increased secretion of CXCL10 by DCs in the tumor. CXCL10 then induces the tumor infiltration of CD8^+^ T cells. Thus, by enhancing the function of Type 17 T cells, 8–074 treatment increased the secretion of CCL20 in the tumor, promoting tumor infiltration of both CD8^+^ T and MoDC and making the anti-tumor immune response of Type 17 T cells efficient and persistent.

### IL17A and CXCL10 were positively correlated with CD8^+^ T cell tumor infiltration

Clinical studies have shown that IL-17A, the main effector of Type 17 T cells, is associated with tumor-infiltrating CD8^+^ T cells [40]. However, there are few reports regarding IL-17A associated with dendritic cells [40]. Our results showed that in the high expression of *IL17A*, the *ITGAX* (a marker of dendritic cells) gene expression was significantly higher than that of the *IL17A* low expression (*P* < 0.001) (Fig. 8e). We also found that the *XCR1* gene expression was positively associated with the *IL17A* expression (*P* < 0.05) (Fig. 8e). We then analyzed the relationship between the *CXCL10* and *CCL20* gene expression and *CD8A* (CD8^+^ T marker gene) in the TCGA-LUAD database. *CCL20* was significantly positively correlated with *CD8A* (*R* = 0.02, *P* = 0.578). *CXCL10* was significantly positively correlated with *CD8A* (*R* = 0.57, *P* = 2.18e-45) (Fig. 8f). We also analyzed the correlation between the expression of *IL17A* and the expression of *CXCL10* as well as *CCL20* (Fig. 8g). It was found that in the case of high expression of *IL17A*, the expression of *CXCL10* and *CCL20* was significantly higher than when the expression of *IL17A* was low (*P* < 0.001). These results indicate that CXCL10 may play an important role in lung cancer’s immune infiltration regulation, helping to better understand the mechanism of Type 17 T cell and CD8^+^ T cell tumor infiltration in the LLC mouse model.

## Discussion

IL-17 has been reported to be associated with various immune responses [4]. In this study, we found that the RORγt agonist treatment increased intratumoral CD8^+^ T cells and MoDCs by promoting CXCL10. The RORγt agonist promoted Type 17 T cell migration by upregulating CCL20 and CCR6 expression as well as Type 17 T cell tumor infiltration, improving the efficacy of anti-PD-1. Thus, a RORγt agonist could foster a TME that facilitates a stronger tumor-inhibition immune response by promoting cytokine production by Type 17 T cells.

DCs were found to elevate the production of CXCL9 and CXCL10 in an IFN-γ-dependent manner, resulting in T cell infiltration to the tumor in many studies [33]. The exact mechanism underlying the upregulation of CXCR3 ligands CXCL9/10 in Type 17 T cells remains to be clarified. MoDCs are necessary and sufficient to accumulate tumor-specific CD8^+^ T cells in tumors, and the accumulation of DCs is due to the CCL20-CCR6 interaction [41]. Therefore, RORγt agonists may accelerate CCL20 production through signaling to Type 17 T cells to attract DC cells, resulting in an elevation of CXCL10 levels and immune CD8^+^ T cell infiltration in the tumor. This hypothesis was also supported by the observation that Th17 cells could stimulate the expression of the chemokine CCL20 in tumor tissue and promote the migration of DC by CCL20-CCR6 dependence [21, 42]. In the lung carcinoma syngeneic model, we confirmed that RORγt agonist treatment increased intratumoral CD8^+^ T cells and MoDCs through the promotion of CXCL10 as well as promotion of Type 17 T cell migration via upregulation of CCL20 and CCR6 expression.

Immunotherapy has emerged as a potent and effective treatment for multiple cancer types. Although a large and growing number of cancer patients benefit from checkpoint blockade and other immunotherapies, a substantial fraction of patients fail to respond clinically [27, 43]. Prior research in non-small-cell lung carcinoma (NSCLC) has demonstrated that high TIICs, particularly CD8^+^ T cells, correlate with response to anti-PD-1 therapy and predict a good prognosis in many solid cancers [27, 44, 45]. Furthermore, patients with high CXCL9/10 levels were found to have better clinical benefits than patients with low CXCL9/10 levels in many clinical trials [46, 47]. In our study, RORγt agonists enhanced immune activation by augmenting CD8^+^ T cell infiltration and decreasing immunosuppression by reducing Treg cells simultaneously. These findings suggest an effective combination strategy of the RORγt agonist combined with current immunotherapies in cancers.

In addition, numerous studies have found that the characteristic chemokines of Type 17 T cells, such as IL-17A, IL-17F, GM-CSF, and CCL20, recruit T cells, B cells, neutral granulocytes, and macrophages into tumor tissue in various tumor models [6, 11, 38]. The infiltrated immune cells, in turn, produced chemokines, including CCL3, CCL4, CCL5, CXCL9, and CXCL10, responsible for the attraction of CD8^+^ T cells and additional neutrophils [21, 38]. Accordingly, we reveal a novel mechanism in which tumor-infiltrating cells, including Type 17 cells, MoDCs, and CD8^+^ T, form an auto-enhancing loop to promote antitumor activity.

IL-17 cytokines have been reported to be double-edged agents and, depending on the type of cancer, can be anti- and pro-tumor cytokines [48]. In our study using the LCC models, we found the anti-tumor effect of the Type T cell was associated with tumor infiltrating CD8^+^ T cells. Our data also suggested a substantial correlation between IL-17A and CD8^+^ T cell infiltration in LLC tumors and indicated a probable mechanism of the indirect anti-tumor effect of Type 17 T cells (Fig. 1c). CD8^+^ T cell depletion via anti-CD8 reduced the overall efficacy of the tumor growth inhibition mediated by 8-074 (Fig. 4d-e). Furthermore, in the ADT model, Tc17 cells reduced tumor growth as well as enhanced tumor infiltration of CXCL10^+^ MoDCs and CD8^+^ T cells (Fig. 8d), suggesting that the antitumor effect of Tc17 in vivo was not only from a direct cytotoxic killing effect of Tc17, but also from the recruitment of CD8 + T cells. The antitumor activity of Type 17 T cells and IL-17A was associated with increased CD8 + T cell tumor infiltration.

RORγ agonist LYC-55716 is being tested in the clinic for advanced or metastatic cancer in a Phase I/II trial (NCT03396497), and in combination with pembrolizumab for NSCLC in a Phase I trial (NCT02929862) [19]. Thus, the discovery and application of RORγt agonist targeting Type 17 T cells will create next-generation cancer immunotherapies. In addition, 8–074 demonstrated improved efficacy both in vitro and in vivo and better selectivity in B cells compared with LYC-55716. Thus, 8–074 could have more promising clinical applications and a better therapeutic window than LYC-55716.

High expression of RORγt is associated with better cancer patient survival in lung cancer and breast cancer, esophageal adenocarcinoma, hepatocellular liver carcinoma, renal clear cell carcinoma, kidney renal clear cell carcinoma, and sarcoma in TCGA (Fig. 1a, Fig. S1a). Our result indicated that RORγt agonists might have broad clinical implications in various tumors such as breast carcinoma, hepatocellular liver carcinoma, and kidney renal clear cell carcinoma.

The main limitation of our study is that the novel mechanism of Type 17 T cells we found in LLC tumor is not observed in other cancer models, and we should compare the immune infiltration between cancers that respond to anti-PD-1 differently. Furthermore, the direct association between Type 17 T cells producing CCL20 and CXCL10^+^ MoDCs is unclear. Finally, some humanized models should be developed to bridge the mechanisms discovered in murine cancer models with the bioinformatics analysis of patient samples.

To the best of our knowledge, this is the first report of a specific mechanism of Type 17 T cells modulating the TME. Understanding how the RORγt agonist enhances immune activity by infiltrating TIICs and promoting the expression of cytokines associated with the TIICs in tumor tissues is crucial for effective tumor inhibition. Cancer immunotherapy may benefit from discovering and applying potent and selective RORγt agonists targeting Type 17 T cells.

## Conclusions

In this study, we reported a novel synthetic RORγt agonist named 8-074 that selectively targets RORγt and can enhance the differentiation of both murine Type 17 T cells and human Type 17 T cells. In addition, RORγt agonists enhanced the antitumor activity of Tc17 cells in vitro in the adopted T cell transfer model. Moreover, we demonstrated that the RORγt agonist treatment induced robust antitumor effects in various tumor models. Infiltration of IFN-γ^+^ CD8^+^ T cells were upregulated in RORγt agonist-treated tumors, a process that was mediated by CXCL10 produced by MoDCs in LCC and MC38 models. The increased numbers of DCs within the tumor were related to CCL20, which is a signature chemokine of Type 17 T cells (Fig. 8h). Finally, a combination of 8-074 with anti-PD-1 provided better efficacy than either single agent alone.

## Supplementary Information

**Additional file 1: Fig. S1.** Identification of survival and immune infiltration *via* the *RORC* pathway in solid cancers. (a) High expression of *RORC* correlated with better prognosis in patients with various cancers. Kaplan-Meier survival curves for patients with BC (Breast Cancer), EAC (Esophageal Adenocarcinoma), KIRC (Kidney renal clear cell carcinoma), and LIHC (Liver hepatocellular carcinoma) using TCGA samples. (b) The mRNA level of *PDCD1* was negatively correlated with *RORC* expression in BRCA (Breast invasive carcinoma), COAD (Colon adenocarcinoma), and LUSC (Lung squamous cell carcinoma). For *RORC* and *PDCD1* expression analysis, we downloaded log2-transformed, normalized mRNA expression values (RSEM, Illumina HiSeq_RNASeqV2) and clinicopathological TCGA cohort data from the Cell Index Database CELLX. (c) The correlation between *CD8A* expression and *IL17A* expression in BRCA (Breast invasive carcinoma), LIHC (Liver hepatocellular carcinoma), and OV (Ovarian serous cystadenocarcinoma) patients in the TCGA database. (d) *IL17A* expression relationship with CD8^+^ T cells in LUAD. TCGA data using TIMER analysis. (e) 2D diagram of molecular docking between LYC-55716 and RORγt agonist crystals (left); and between 8-074 and RORγt agonist crystals (right). Data is shown as the mean ± SD from a representative experiment, and a Student's t-test was used for determining significance. Experiments were repeated three times with consistent results.

**Additional file 2: Fig. S2**. Selectivity of RORγt agonists *in vitro*. (a) The activity of 8-074 in Gal4 reporter gene assays with RORα and RORβ. (RORα EC50 > 10 μM, RORβ EC50 > 10 μM). (b) Summary of bioactivity and selectivity profiles for 8-074 using multiple assays. (c) 8-074 treated Type 17 T cells secreted more Th17 signature cytokines. IL-17F and IL-22 levels secreted by Type 17 T cells during differentiation were assayed by ELISA (*P < 0.05, **P < 0.01). (d) CCK-8 assay was used to determine the cell viability of EL4 cells after treating them with different 8-074 concentrations. Representative data are shown from three independent experiments. (e) Statistical results of apoptosis assays based on FCM as a measure of apoptotic EL4 cells. (f) Representative flow graph. Toxicity evaluation of 8-074 *in vitro*. Lymphoma EL4 cells were treated with various concentrations of 8-074 for 48 hours and analyzed by flow cytometry after Annexin V-FITC/PI staining. (g) Representative flow graph of Th1 cells in a CD4^+^ T population. (h) The relative mRNA expression of *Cd19* in B cells as determined by qPCR. Toxicity evaluation of 8-074 *in vivo*. (i) Relative mRNA expression of *Il-1β* levels in macrophages as determined by qPCR. ***P < 0.001. (j) Relative mRNA expression of *Il-6* in macrophages as determined by qPCR. *P < 0.05. (k) Relative mRNA expression of *Il-10* in macrophages as determined by qPCR. *P < 0.05. Data are shown as mean ± SD from a representative experiment, and Student's t-test was used for determining statistical significance. Experiments were repeated three times with consistent results.

**Additional file 3: Fig. S3.** Selectivity and safety analysis of 8-074. (a) Representative hematoxylin and eosin (H&E) staining micrograph (200 ×) of heart, liver, lung, kidney, spleen, and intestine sections from mice receiving vehicle, 50 mg/kg, or 100 mg/kg 8-074 for two weeks. Scale bar = 100 μm. Vehicle, daily administration of 50 μL DMSO by intraperitoneal injection; 50 mg/kg and 100 mg/kg, daily administration of the corresponding dose (volume less than or equal to 50 μL) by intraperitoneal injection. The cell morphology, number, and distribution in heart, liver, lung, kidney, spleen, and intestine tissues after 8-074 injection were not different from those in the vehicle group. (b and c) Effects of 8-074 on body weight in mice. According to the data after treatment, LLC mice tumor volume changes are shown in Fig.3a and 3b. Data represents the mean ± SD from biological quadruplicates. All error bars represent mean ± SD. Data are from three independent experiments.

**Additional file 4: Fig. S4. **Flow cytometry analysis of 8-074 treated tumors. (a) FACS analysis of Treg from LLC tumor-bearing mice in Figure 3B for the vehicle group and 8-074 (50 mg/kg) group. Representative flow panels from CD45^+^ CD3^+^ CD4^+^ CD8^-^ FOXP3^+^ T cells (Treg) are shown. (b) Statistical results of FCM analysis in LLC tumors. The ratio of Treg in the CD3^+^ cell populations and the CD8/Treg ratio in LLC tumors are shown (***P < 0.001 and ****P < 0.0001). (c) Relative mRNA expression of *Ifn-γ* in LLC tumors as determined by qPCR. N = 3 per group, **P < 0.01, by Student’s t-test. (d) ELISA assayed IFN-γ levels in LLC tumors (N = 3 per group, **P < 0.01, ***P < 0.001, by Student’s t-test). Data shown are mean ± SD of tumor volume for each group (N = 4 – 5 per group, *P < 0.05, **P < 0.01, *** *P* < 0.001 and **** *P* < 0.0001, by Student’s t-test). Data represents the mean ± SD from biological quadruplicates. All error bars represent the mean ± SD. Data are from three independent experiments.

**Additional file 5: Fig. S5.** Molecular mechanism of 8-074 treated Th17 and Tc17 cells. (a) Representative flow panels from an analysis of CXCL10^+^ DCs in MC38 tumors. (b) mRNA level of *Ccl20* in Tc17 cells with or without 8-074 as analyzed by qPCR. Student’s t-test was used for statistical testing (**P < 0.01, ****P < 0.0001). (c) Protein level of CCL20 in Th17 and Tc17 cells with or without 8-074 as analyzed by ELISA. Student’s t-test was used for determining statistical significance (**P < 0.01). Data are shown as the mean ± SD from a representative experiment, and a Student's t-test was used for statistical significance. Experiments were repeated three times with consistent results.

**Additional file 6: Table S1.** Primers used in Quantitative PCR.

**Additional file 7: Table S2.** Preclinical C57 Mouse PK Study Report.

## Data Availability

Public Data Resources: The TCGA datasets, including COAD and READ, were downloaded from cBioPortal (http://www.cbioportal.org/). Other data that supported the findings of this study are available upon request. Data is available at: https://figshare.com/s/323f1ae14d0b244d1964

## Abbreviations

ADT: Adoptive transfer assay
APCs: Antigen-presenting cells
BRCA: Breast invasive carcinoma
CCL20: Chemokine (C-C motif) ligand 20
CCR6: C-C Motif Chemokine Receptor 6
CTL: Cytotoxic T lymphocytes
CXCL10: CXCchemokineligand-10
dual FRET: Dual fluorescent resonance energy transfer
IL-17: Interleukin 17
LBD: Ligand binding domain
LIHC: Liver hepatocellular carcinoma
LLC: Lewis lung carcinoma
MoDCs: Monocyte-derived dendritic cells
NK: Natural killer cells
NSCLC: Non-small-cell lung carcinoma
OV: Ovarian serous cystadenocarcinoma
RORγt: Retinoic acid-related orphan receptor γt
SP: The standard precision
Tc17: Cytotoxic T cell 17
TCGA: The Cancer Genome Atlas
TCR: T cell receptor
TDLN: Tumor-draining lymph node
TGI: Tumor growth inhibition
TIICs: Tumor-infiltrating immune cells
Th17: T helper cell 17
TME: Tumor microenvironment
