# Supplementary figures and images for: RORγt agonist enhances anti-PD-1 therapy by promoting monocyte-derived dendritic cells through CXCL10 in cancers
Source: J Exp Clin Cancer Res. 2022 Apr 23;41:155. doi: 10.1186/s13046-022-02289-2 (PMC9034499; doi:10.1186/s13046-022-02289-2)

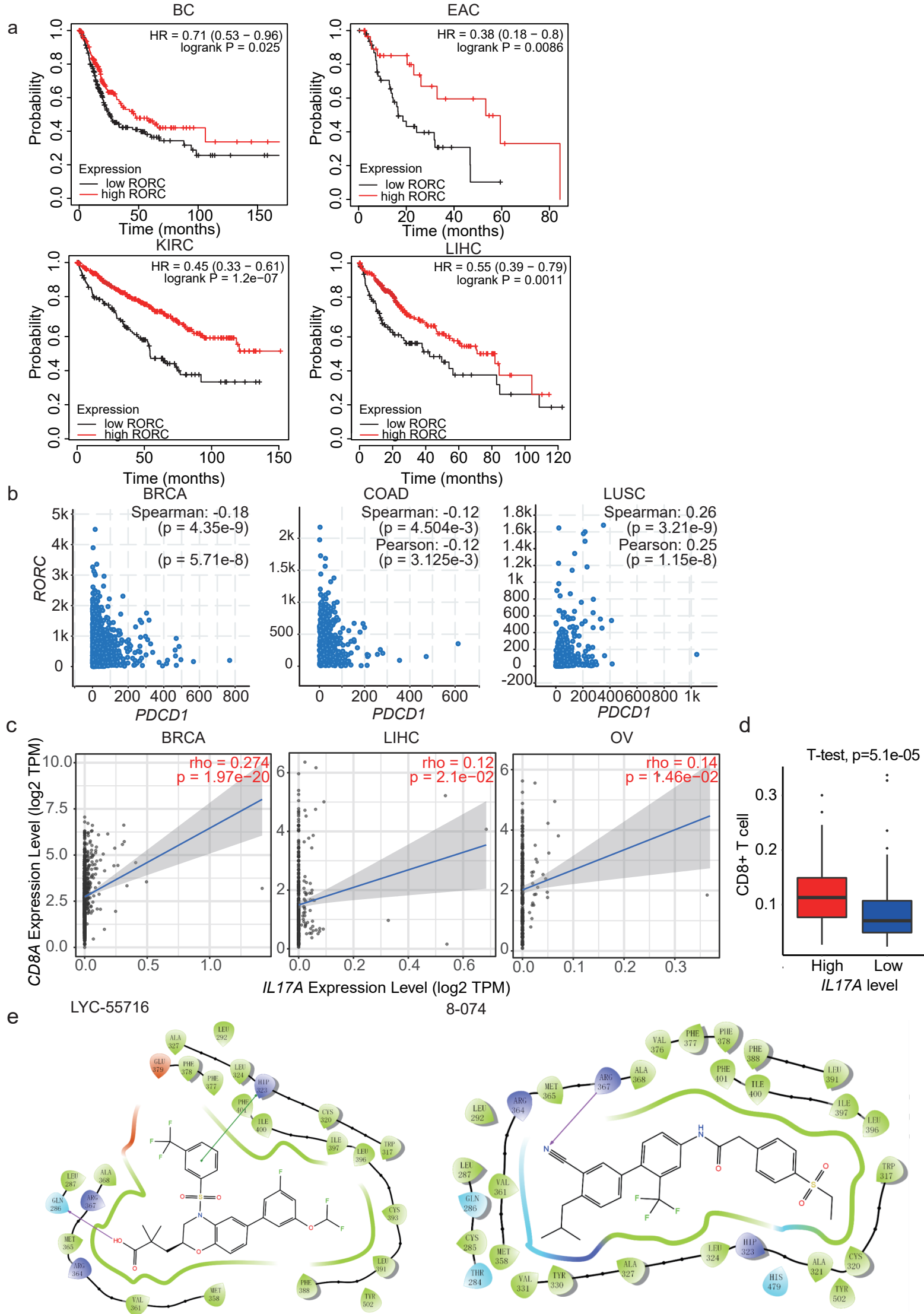

Supplement: Supplementary file 1 — Additional file 1: Fig. S1. Identification of survival and immune infiltration via the RORC pathway in solid cancers. (a) High expression of RORC correlated with better prognosis in patients with various cancers. Kaplan-Meier survival curves for patients with BC (Breast Cancer), EAC (Esophageal Adenocarcinoma), KIRC (Kidney renal clear cell carcinoma), and LIHC (Liver hepatocellular carcinoma) using TCGA samples. (b) The mRNA level of PDCD1 was negatively correlated with RORC expression in BRCA (Breast invasive carcinoma), COAD (Colon adenocarcinoma), and LUSC (Lung squamous cell carcinoma). For RORC and PDCD1 expression analysis, we downloaded log2-transformed, normalized mRNA expression values (RSEM, Illumina HiSeq_RNASeqV2) and clinicopathological TCGA cohort data from the Cell Index Database CELLX. (c) The correlation between CD8A expression and IL17A expression in BRCA (Breast invasive carcinoma), LIHC (Liver hepatocellular carcinoma), and OV (Ovarian serous cystadenocarcinoma) patients in the TCGA database. (d) IL17A expression relationship with CD8+ T cells in LUAD. TCGA data using TIMER analysis. (e) 2D diagram of molecular docking between LYC-55716 and RORγt agonist crystals (left); and between 8-074 and RORγt agonist crystals (right). Data is shown as the mean ± SD from a representative experiment, and a Student's t-test was used for determining significance. Experiments were repeated three times with consistent results. [file 13046_2022_2289_MOESM1_ESM.pdf]

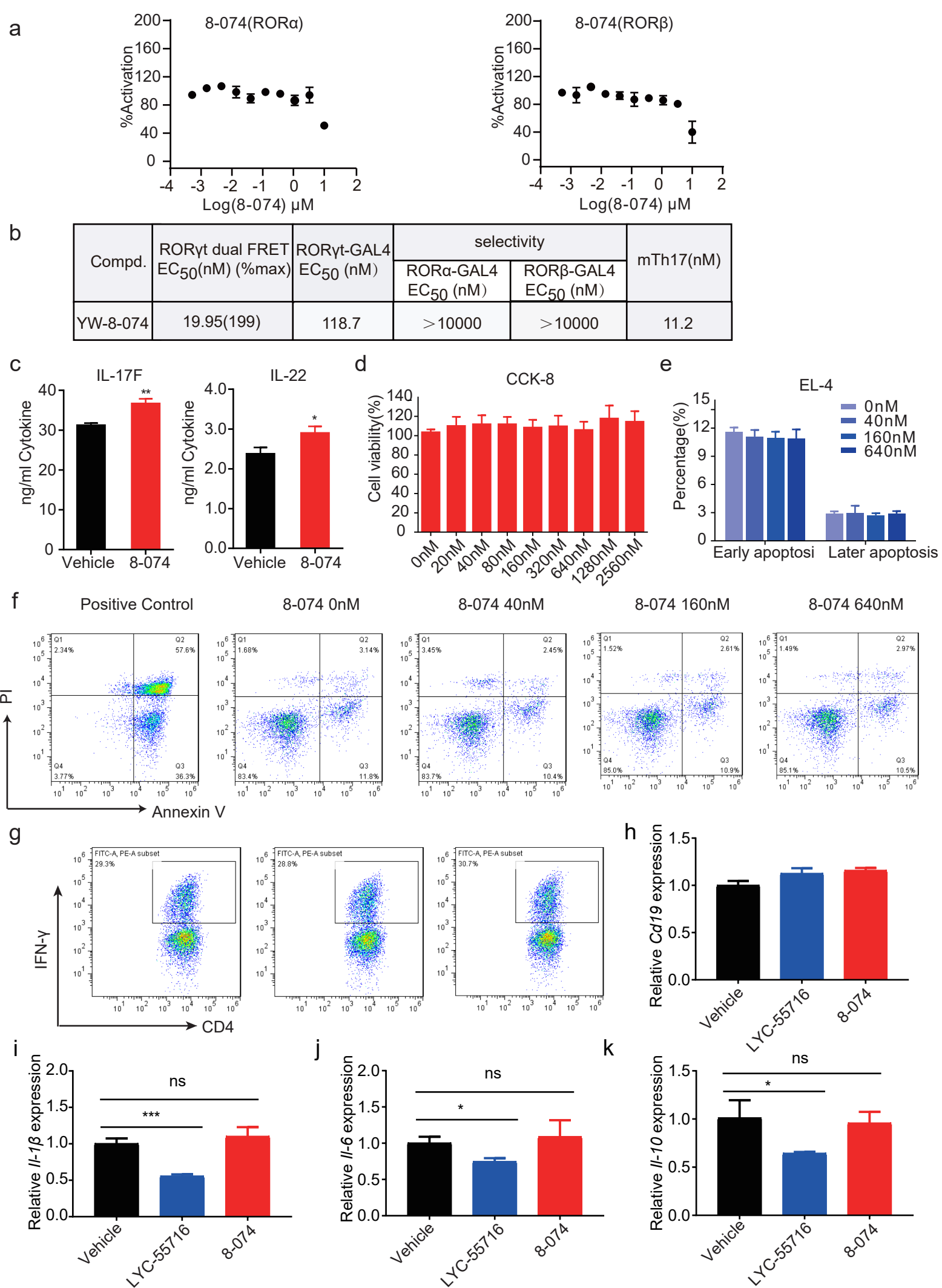

Supplement: Supplementary file 2 — Additional file 2: Fig. S2. Selectivity of RORγt agonists in vitro. (a) The activity of 8-074 in Gal4 reporter gene assays with RORα and RORβ. (RORα EC50 > 10 μM, RORβ EC50 > 10 μM). (b) Summary of bioactivity and selectivity profiles for 8-074 using multiple assays. (c) 8-074 treated Type 17 T cells secreted more Th17 signature cytokines. IL-17F and IL-22 levels secreted by Type 17 T cells during differentiation were assayed by ELISA (*P < 0.05, **P < 0.01). (d) CCK-8 assay was used to determine the cell viability of EL4 cells after treating them with different 8-074 concentrations. Representative data are shown from three independent experiments. (e) Statistical results of apoptosis assays based on FCM as a measure of apoptotic EL4 cells. (f) Representative flow graph. Toxicity evaluation of 8-074 in vitro. Lymphoma EL4 cells were treated with various concentrations of 8-074 for 48 hours and analyzed by flow cytometry after Annexin V-FITC/PI staining. (g) Representative flow graph of Th1 cells in a CD4+ T population. (h) The relative mRNA expression of Cd19 in B cells as determined by qPCR. Toxicity evaluation of 8-074 in vivo. (i) Relative mRNA expression of Il-1β levels in macrophages as determined by qPCR. ***P < 0.001. (j) Relative mRNA expression of Il-6 in macrophages as determined by qPCR. *P < 0.05. (k) Relative mRNA expression of Il-10 in macrophages as determined by qPCR. *P < 0.05. Data are shown as mean ± SD from a representative experiment, and Student's t-test was used for determining statistical significance. Experiments were repeated three times with consistent results. [file 13046_2022_2289_MOESM2_ESM.pdf]

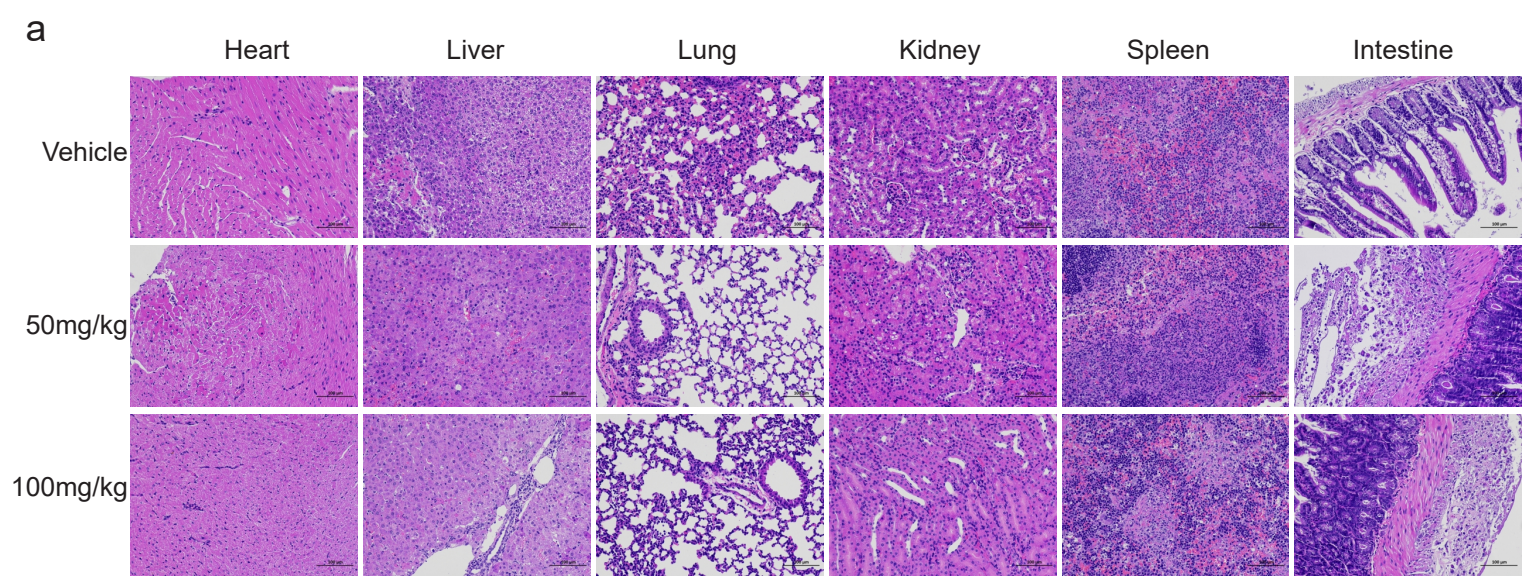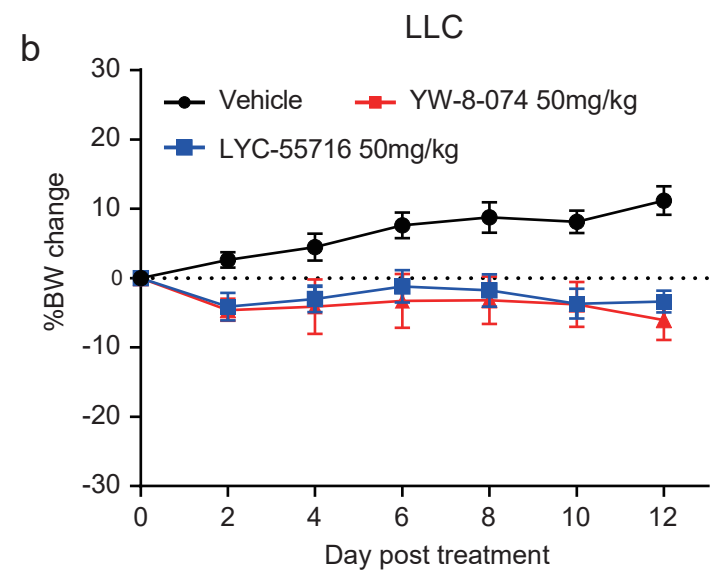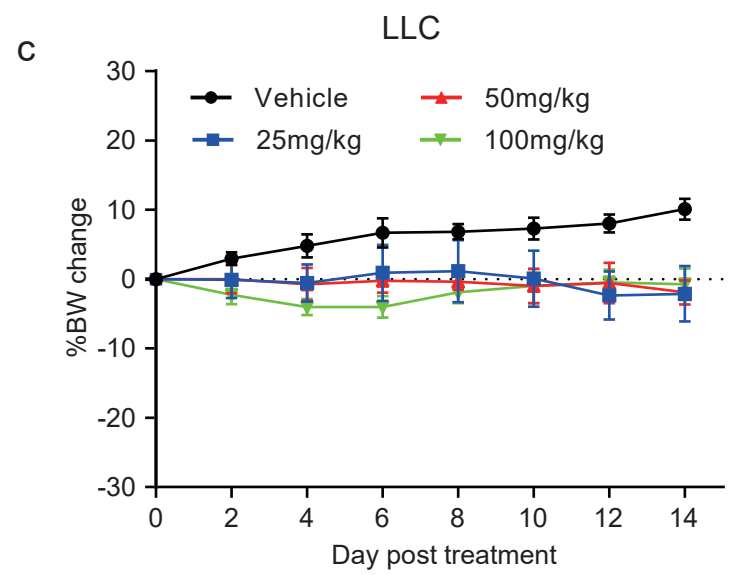

Supplement: Supplementary file 3 — Additional file 3: Fig. S3. Selectivity and safety analysis of 8-074. (a) Representative hematoxylin and eosin (H&E) staining micrograph (200 ×) of heart, liver, lung, kidney, spleen, and intestine sections from mice receiving vehicle, 50 mg/kg, or 100 mg/kg 8-074 for two weeks. Scale bar = 100 μm. Vehicle, daily administration of 50 μL DMSO by intraperitoneal injection; 50 mg/kg and 100 mg/kg, daily administration of the corresponding dose (volume less than or equal to 50 μL) by intraperitoneal injection. The cell morphology, number, and distribution in heart, liver, lung, kidney, spleen, and intestine tissues after 8-074 injection were not different from those in the vehicle group. (b and c) Effects of 8-074 on body weight in mice. According to the data after treatment, LLC mice tumor volume changes are shown in Fig.3a and 3b. Data represents the mean ± SD from biological quadruplicates. All error bars represent mean ± SD. Data are from three independent experiments. [file 13046_2022_2289_MOESM3_ESM.pdf]

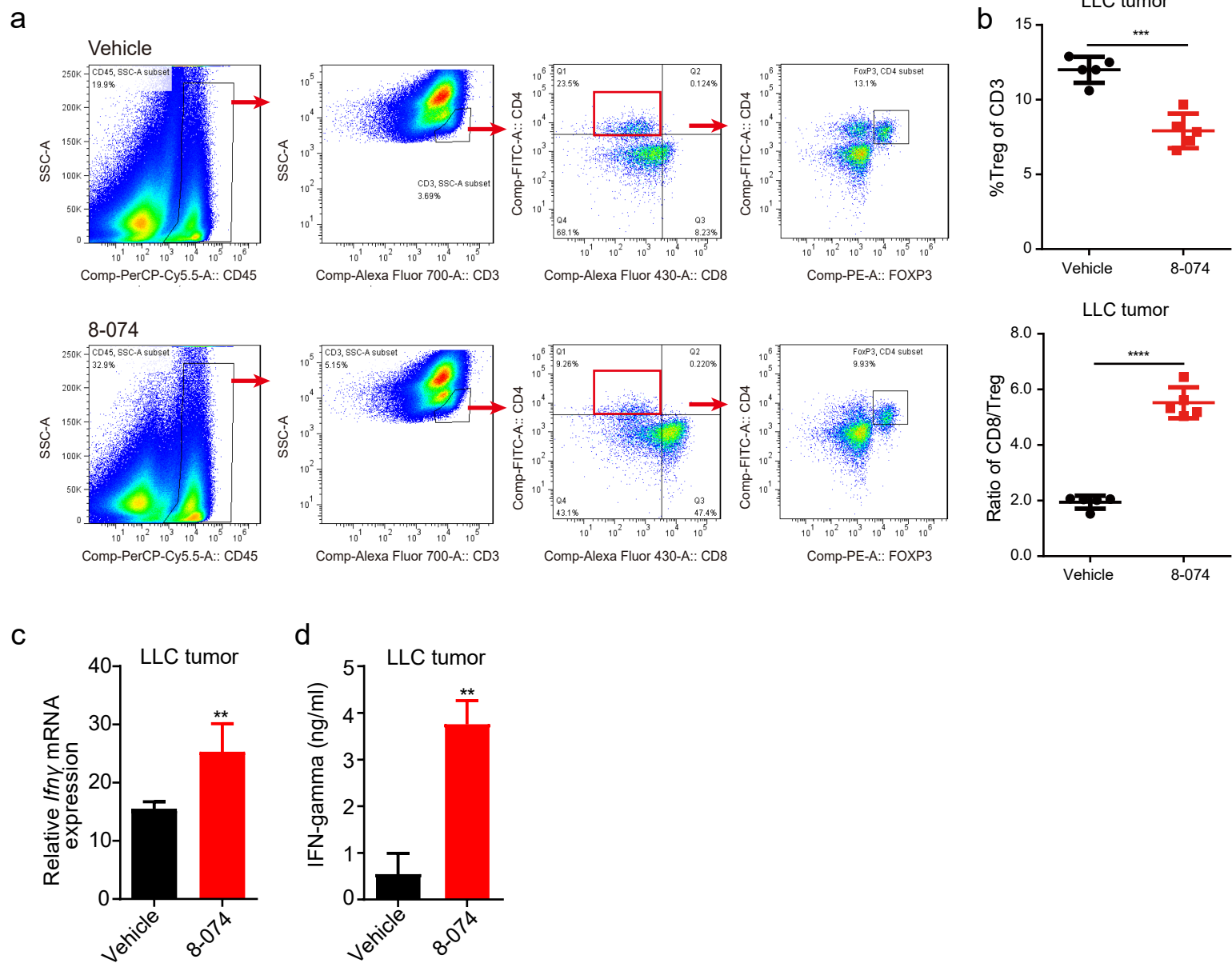

Supplement: Supplementary file 4 — Additional file 4: Fig. S4. Flow cytometry analysis of 8-074 treated tumors. (a) FACS analysis of Treg from LLC tumor-bearing mice in Figure 3B for the vehicle group and 8-074 (50 mg/kg) group. Representative flow panels from CD45+ CD3+ CD4+ CD8- FOXP3+ T cells (Treg) are shown. (b) Statistical results of FCM analysis in LLC tumors. The ratio of Treg in the CD3+ cell populations and the CD8/Treg ratio in LLC tumors are shown (***P < 0.001 and ****P < 0.0001). (c) Relative mRNA expression of Ifn-γ in LLC tumors as determined by qPCR. N = 3 per group, **P < 0.01, by Student’s t-test. (d) ELISA assayed IFN-γ levels in LLC tumors (N = 3 per group, **P < 0.01, ***P < 0.001, by Student’s t-test). Data shown are mean ± SD of tumor volume for each group (N = 4 – 5 per group, *P < 0.05, **P < 0.01, *** P < 0.001 and **** P < 0.0001, by Student’s t-test). Data represents the mean ± SD from biological quadruplicates. All error bars represent the mean ± SD. Data are from three independent experiments. [file 13046_2022_2289_MOESM4_ESM.pdf]

**a**

Vehicle

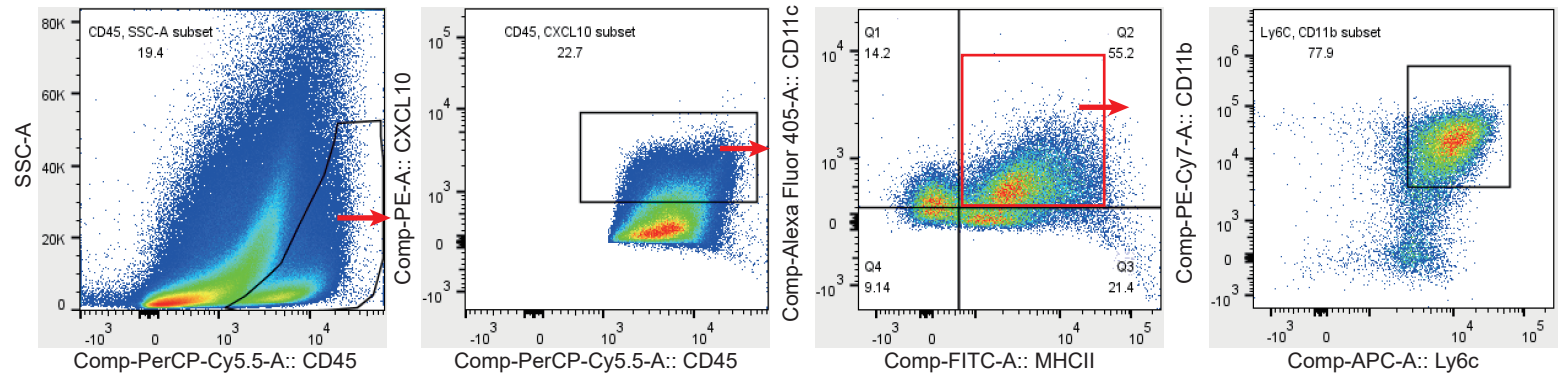

8-074

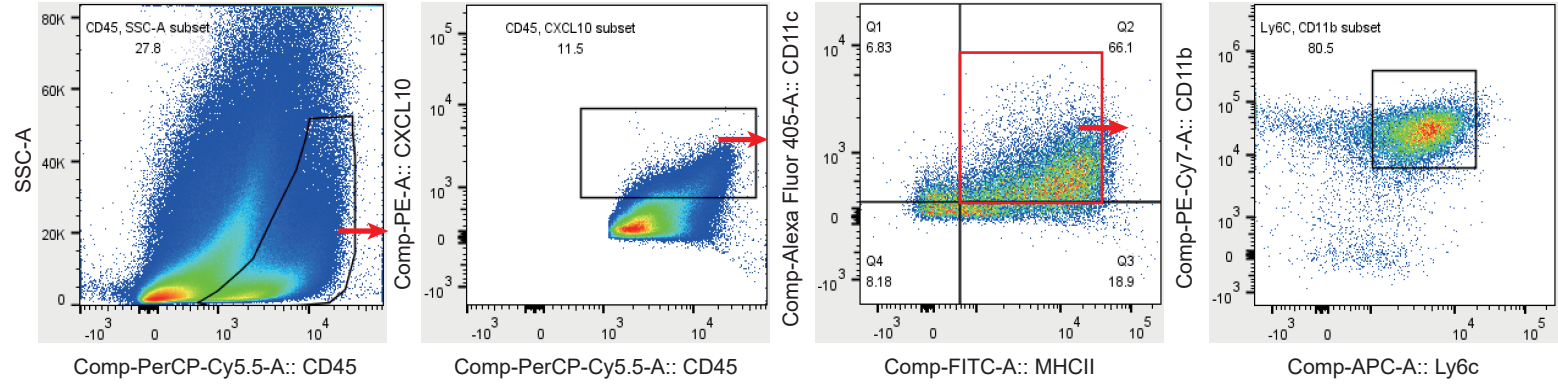**b**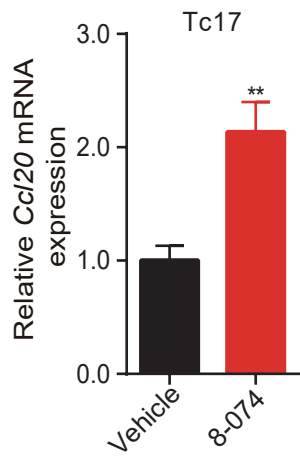**c**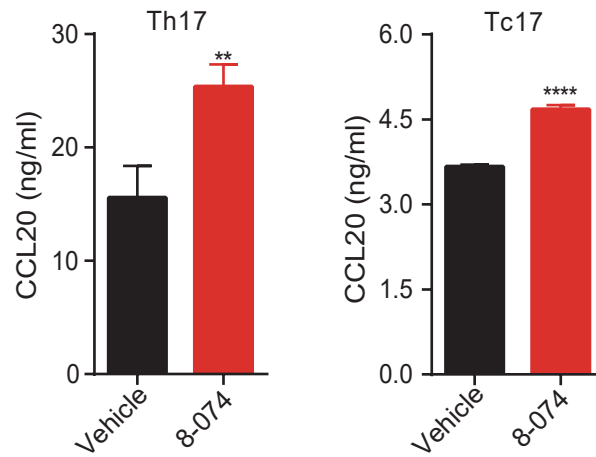

Supplement: Supplementary file 5 — Additional file 5: Fig. S5. Molecular mechanism of 8-074 treated Th17 and Tc17 cells. (a) Representative flow panels from an analysis of CXCL10+ DCs in MC38 tumors. (b) mRNA level of Ccl20 in Tc17 cells with or without 8-074 as analyzed by qPCR. Student’s t-test was used for statistical testing (**P < 0.01, ****P < 0.0001). (c) Protein level of CCL20 in Th17 and Tc17 cells with or without 8-074 as analyzed by ELISA. Student’s t-test was used for determining statistical significance (**P < 0.01). Data are shown as the mean ± SD from a representative experiment, and a Student's t-test was used for statistical significance. Experiments were repeated three times with consistent results. [file 13046_2022_2289_MOESM5_ESM.pdf]
