# Supplementary material for: RORγt agonist enhances anti-PD-1 therapy by promoting monocyte-derived dendritic cells through CXCL10 in cancers
Source: J Exp Clin Cancer Res. 2022 Apr 23;41:155. doi: 10.1186/s13046-022-02289-2 (PMC9034499; doi:10.1186/s13046-022-02289-2)
Supplement: Supplementary file 6 — Additional file 6: Table S1. Primers used in Quantitative PCR. [file 13046_2022_2289_MOESM6_ESM.docx]

**Additional file 6: Table S1**

Primers used in Quantitative PCR

| Gene | sense primer (5’-3’) | Anti-sense primer (5’-3’) |
| --- | --- | --- |
| *Il-17a* | CCCTCAGACTACCTCAACCGTTC | TCATGTGGTGGTCCAGCTTTCC |
| *Il-17f* | ACCAGGGCATTTCTGTCCCAC | GACGGAGTTCATGGTGCTGTCTT |
| *Gmcsf* | CATCAAAGAAGCCCTGAACCTC | CGTAGACCCTGCTCGAATATCTTC |
| *Rorc* | CCGCAGCCAGCAGTGTAATGT | GCTTCTTGGACATTCGGCCAAAC |
| *Il-23a* | AATAATGTGCCCCGTATCCAGT | CTATCAGGGAGTAGAGCAGGC |
| *Il-23r* | GGTCCAAGCTGTCAATTCCCTA | CAGTATCGTTTGTAGTCTCAGCCC |
| *Il-21* | AGACATTCATCATTGACCTCGTGG | CATACGAATCACAGGAAGGGCA |
| *Rora* | CCCCTACTGTTCCTTCACCAA | AAGGTCTGCCACGTTATCTGC |
| *Tcra* | CAGCAGCAGGTGAGACAAAGT | GGCTTTATAATTAGCTTGGTCC |
| *Actb* | GGGAAATCGTGCGTGACATCA | TCATGGATGCCACAGGATTCC |
| *Gapdh* | GAGTGTTTCCTCGTCCCGTAG | TCGCTCCTGGAAGATGGTGAT |
| *Cd8a* | GACCTGGTATGTGAAGTGTTGG | TTATCTTGTTGTGGGATGAAGC |
| *Cxcl10* | ATATCGATGACGGGCCAGTGAG | TCGTGGCAATGATCTCAACACG |
| *Ccr6* | TTTGTCCTCACCCTACCGTTCT | GGAGCAGCATCCCACAGTTAA |
| *Ccl20* | TATCTGCTGCCAGGACTACATCC | CTTCTTCACCCAGACCTGCCT |
| *Cd86* | CAGCACGGACTTGAACAACC | CTCCACGGAAACAGCATCTGA |
| *Cd19* | CGTGGAGGATAGTGGGGAGATG | CACACATACAGCTGGGAACCA |
| *Il-1β* | TGACGTTCCCATTAGACAACTG | CCGTCTTTCATTACACAGGACA |
| *IL-6* | ACAAGTCCGGAGAGGAGACT | TTGCCATTGCACAACTCTTTTC |
| *IL-10* | CCGCTTCATCCCTGAAAACTG | AGATGCTGCTACAAAGGCAGA |
| *Arg1* | CGTAGACCCTGGGGAACACT | CGGCCTTTTCTTCCTTCCCAG |
