# Supplementary material for: RORγt agonist enhances anti-PD-1 therapy by promoting monocyte-derived dendritic cells through CXCL10 in cancers
Source: J Exp Clin Cancer Res. 2022 Apr 23;41:155. doi: 10.1186/s13046-022-02289-2 (PMC9034499; doi:10.1186/s13046-022-02289-2)
Supplement: Supplementary file 7 — Additional file 7: Table S2. Preclinical C57 Mouse PK Study Report. [file 13046_2022_2289_MOESM7_ESM.docx]

**Additional file 7: Table S2**

Preclinical C57 Mouse PK Study Report

| Dosing Route | i.v. | i.g. |
| --- | --- | --- |
| Dose (mg/kg) | 2 | 5 |
| Tmax (h) | 0.25 ± 0 | 1.67 ± 0.58 |
| Cmax (ng/ml) | 4800 ± 1200 | 3020 ± 110 |
| AUClast (h*ng/ml) | 20,500 ± 1500 | 19,700 ± 9300 |
| AUCINF (h*ng/ml) | 20,600 ± 1500 | 19,800 ± 9400 |
| MRTINF (h) | 3.84 ± 0.33 | 4.49 ± 1.2 |
| MRTINF (h) | 2.97 ± 0.25 | 3.34 ± 0.52 |
| Rsq | 0.97 ± 0.0032 | 0.968 ± 0.025 |
| Cl (ml/min/kg) | 1.63 ± 0.11 | 38.3 ± 18.1 |
